# Regulatory role of cathepsin L in induction of nuclear laminopathy in Alzheimer’s disease

**DOI:** 10.1111/acel.13531

**Published:** 2021-12-14

**Authors:** Md Imamul Islam, Pandian Nagakannan, Tetiana Shcholok, Fabio Contu, Sabine Mai, Benedict C Albensi, Marc R. Del Bigio, Jun‐Feng Wang, Md Golam Sharoar, Riqiang Yan, Il‐Seon Park, Eftekhar Eftekharpour

**Affiliations:** ^1^ Department of Physiology and Pathophysiology University of Manitoba Winnipeg MB Canada; ^2^ Rady Faculty of Health Sciences University of Manitoba Winnipeg MB Canada; ^3^ Cell Biology Research Institute of Oncology and Hematology CancerCare Manitoba University of Manitoba Winnipeg MB Canada; ^4^ St Boniface Hospital Albrechtsen Research Centre Winnipeg MB Canada; ^5^ Department of Pharmaceutical Sciences College of Pharmacy Nova Southeastern University Fort Lauderdale Florida USA; ^6^ Department of Pathology Shared Health Manitoba University of Manitoba Winnipeg MB Canada; ^7^ Department of Pharmacology and Therapeutics University of Manitoba Winnipeg MB Canada; ^8^ Department of Neuroscience University of Connecticut Health Farmington Connecticut USA; ^9^ Department of Cellular and Molecular Medicine Chosun University Gwangju South Korea

**Keywords:** acetylation, amyloid beta, chromatin, histone, lysosomal membrane permeabilization, methylation, nuclear lamina, super‐resolution microscopy

## Abstract

Experimental and clinical therapies in the field of Alzheimer's disease (AD) have focused on elimination of extracellular amyloid beta aggregates or prevention of cytoplasmic neuronal fibrillary tangles formation, yet these approaches have been generally ineffective. Interruption of nuclear lamina integrity, or laminopathy, is a newly identified concept in AD pathophysiology. Unraveling the molecular players in the induction of nuclear lamina damage may lead to identification of new therapies. Here, using 3xTg and APP/PS1 mouse models of AD, and in vitro model of amyloid beta42 (Aβ42) toxicity in primary neuronal cultures and SH‐SY5Y neuroblastoma cells, we have uncovered a key role for cathepsin L in the induction of nuclear lamina damage. The applicability of our findings to AD pathophysiology was validated in brain autopsy samples from patients. We report that upregulation of cathepsin L is an important process in the induction of nuclear lamina damage, shown by lamin B1 cleavage, and is associated with epigenetic modifications in AD pathophysiology. More importantly, pharmacological targeting and genetic knock out of cathepsin L mitigated Aβ42 induced lamin B1 degradation and downstream structural and molecular changes. Affirming these findings, overexpression of cathepsin L alone was sufficient to induce lamin B1 cleavage. The proteolytic activity of cathepsin L on lamin B1 was confirmed using mass spectrometry. Our research identifies cathepsin L as a newly identified lamin B1 protease and mediator of laminopathy observed in AD. These results uncover a new aspect in the pathophysiology of AD that can be pharmacologically prevented, raising hope for potential therapeutic interventions.

## Graphical abstract

Aβ42 works by binding to cell membrane, interacting with receptors, forming channel‐like structure or by directly entering into the cells to interact intracellular targets. Treatment of Aβ42 did not induce apoptosis, rather induced lysosomal membrane permeabilization leading to release of lysosomal cathepsins. Cathepsin L cleaves nuclear LB1 and eventually evokes nuclear deformation/invagination. This results in exposing lamin‐associated histones susceptible for post‐translational modifications. The consequences of histone modifications deserve further studies.

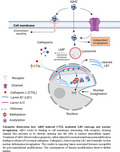

**Abbreviations**
AD: Alzheimer's disease
Aβ42: amyloid beta42
CASP: caspase
CTSB: cathepsin B
CTSD: cathepsin D
CTSL: cathepsin L
LB1: Lamin B1
MEF: mouse embryonic fibroblast
NL: nuclear lamina

## 1 INTRODUCTION

Alzheimer's disease (AD) accounts for ~64% of all dementias (Long & Holtzman, 2019). The pathophysiology of the disease is highly complex and includes neuronal and glial cells functional deficits. These include the disruption of oxidative stress management, defective proteostasis systems, and accumulation of amyloidogenic processing of amyloid precursor protein and hyperphosphorylation of Tau (Arranz & De Strooper, 2019; Hohn et al., 2020). These result in deposition of extracellular amyloid beta (Aβ) and intracellular accumulation of neurofibrillary tangles (NFTs) and trigger the progressive neuronal degeneration, causing memory loss and eventual patient's death within 5–12 years of diagnosis (Duyckaerts et al., 2009; Hardy & Higgins, 1992; Jack et al., 2018). Despite extensive basic research and clinical trials during the last four decades, there are currently no effective standard treatments that can prevent the loss of neurons in this disease. Although some positive news on potential new therapies including Aducanumab targeting Aβ or anti‐Tau antibodies have raised some hope (Vaz & Silvestre, 2020), the need for understanding the mechanisms of neuronal death in AD for finding more effective therapies retains its utmost priority.

Neuronal laminopathy is a newly identified concept in the pathophysiology of AD. Nuclear lamina (NL) is a dense fibrillar protein layer that is located at the interface of nuclear envelope inner layer and chromatin. NL undergoes significant changes during cell division, proliferation, and differentiation as well as in different pathological conditions (Broers et al., 2006). There are two classes of proteins in NL: lamin A/C and lamin B1/B2. Lamin A/C are encoded by alternatively spliced *LMNA* transcripts, but lamin B1 and B2 are encoded by *LMNB1* and *LMNB2*, respectively (Malhas et al., 2009). These proteins play important roles in chromatin stability and gene expression, and mutations/defects in these proteins lead to a class of diseases known as laminopathy. More than 300 mutations in *LMNA* gene have been reported in association with several developmental diseases affecting mesodermal tissues, but sparing the central nervous system (Broers et al., 2006). The sparing has been linked to low levels of lamin A protein in neural cells, which is mediated by microRNA‐9 through downregulation of progerin and prelamin A transcripts (Frost, 2016; Young et al., 2014). B‐type lamins are widely expressed in all types of tissues and provide a tethering function for chromatin, therefore are involved in many vital systems through modulation of gene regulation (Butin‐Israeli et al., 2015). Lamin B1/B2 (LB1/2) also play crucial roles in brain development and neuronal survival under oxidative stress (Chen et al., 2019; Coffinier et al., 2010). Genetic deletion studies of *LMNB1* and *LMNB2* show that LB1 is indispensable in the adult brain, partially due to its regulatory role in the transcription of antioxidant genes, mediated by direct interaction with Oct1, a transcription factor responsible for regulation of antioxidant systems (Malhas et al., 2009).

Nuclear lamina damage in AD was originally described in a model of tauopathy in *Drosophila*, in which genetic inhibition of thioredoxin reductase‐1 (TrxR1) or superoxide dismutase resulted in hyperphosphorylation of Tau and changes in chromatin density. Tauopathy was associated with NL damage‐causing nuclear envelope invagination and aberrant gene expression (Frost et al., 2014). A series of studies in cardiac and neural cells propose the involvement of mechanical stress in induction of laminopathy (Bertero et al., 2019; Frost et al., 2016). In AD, formation of NFTs is believed to be the source of this mechanical stress that causes interruption of NL integrity as shown by LB1 disruption. There seems to be an association between cell death mode and the extent of NL damage; in caspase‐dependent (apoptotic) cell death. NL is separated from the nuclear envelope, while in developmental programmed cell death (caspase independent) NL remains attached to the envelope (Lindenboim et al., 2020). The identity of molecular players causing NL damage, however, remains mostly undetermined.

We recently showed that LB1 is a substrate of caspase‐6 (CASP6) that is activated after diminished thioredoxin (Trx1) reducing capacity (Islam et al., 2019). Increased CASP6 (Halawani et al., 2010; L. Zhou et al., 2019) and decreased Trx1 levels (Lovell et al., 2000; Raffel et al., 2003; Venojarvi et al., 2014) are well‐established hallmarks of AD, indicating the relevance of our findings to pathophysiology of the disease. In addition to CASP6, a wide range of other proteases are known to mediate LB1 degradation. This list includes CASP3, granzyme A and B, as well as nuclear scaffold proteases (Islam et al., 2019; Kivinen et al., 2005; Ramasamy et al., 2016; Zhang et al., 2001). LB1 degradation can also be mediated by phosphorylation of its specific amino acid residues (Chang et al., 2011). Disruption of LB1 integrity is reported as part of autophagy process, proposing a potential role for lysosomal enzymes (Butin‐Israeli et al., 2015). More evidence for lysosomal enzymes involvement in NL damage has been shown by identification of LB‐LC3 conjugates in cellular senescence (Dou et al., 2015); however, the identity of these enzymes has not been determined, which could have valuable therapeutic implications. Building on previous reports on lysosomal deficiency in models of AD (Hung & Livesey, 2018), and the involvement of CASP6 in induction of LB1 degradation (Islam et al., 2019), in this study, we aimed to examine the involvement of lysosomal enzymes in NL damage and assess its potential amenability to pharmacological interventions.

## 2 RESULTS

### 2.1 LB1 degradation in NL damage is associated with upregulation of lysosomal cathepsins in the 3xTg model of AD

**FIGURE 1:**
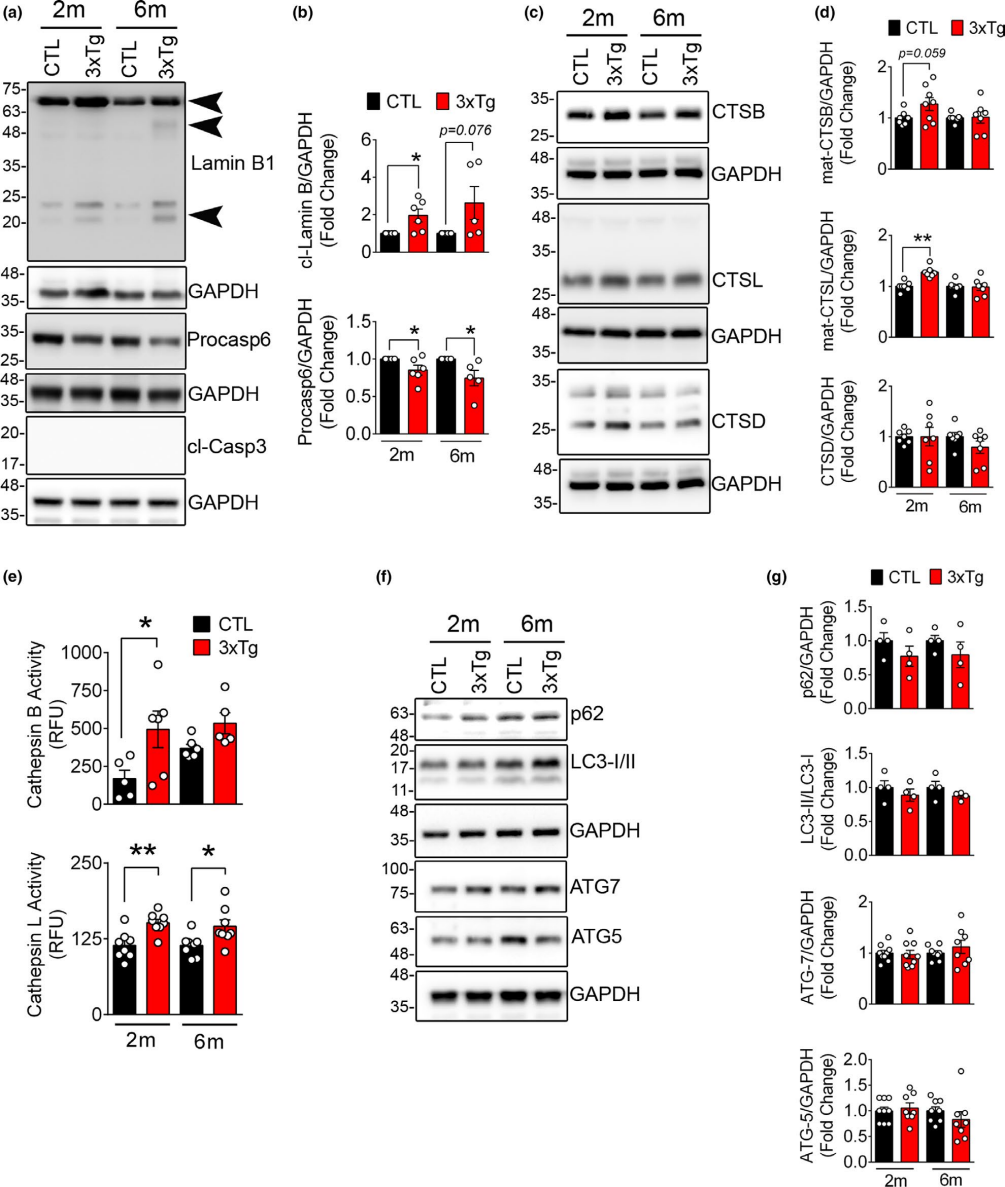
Lamin B1 degradation and upregulation of lysosomal cathepsins in 3xTg mouse hippocampus tissue (a) Representative Western blots showing the expression of lamin B1, pro‐casp6, and cl‐Casp3 in hippocampal lysates from CTL and age‐matched 3xTg mouse. (b) Quantification of cleaved lamin B1 (21 kDa) and pro‐casp6 is shown in 2 months (2m) and 6 months (6m) old mice. (*n* = 5–6 mice/group), data shown as ±SEM, *p < 0.05. Increased cleaved lamin B1 in 3xTg mice is detected by appearance of a 46 kDa and a 21 kDa (arrowhead). As reported previously, a significant decrease in pro‐casp6 protein was confirmed in 3xTg mouse. We did not detect any indication of apoptosis in these mice as assessed by lack of cl‐Casp3. (c) Representative Western blots showing lysosomal cathepsins in 3xTg mouse hippocampus were quantified using densitometry. (d) Bar graph shows increased levels of CTSB and CTSL in 3xTg mouse, although CTSD levels remained unchanged. (e) Enzymatic activity of CTSB and CTSL activity in CTL and age‐matched 3xTg hippocampal lysates. (f) Markers of autophagy progression were assessed using immunoblotting. (g) No significant change in autophagy progression was observed in this model

Tau hyperphosphorylation and NFT formation has been previously shown as a cause of NL damage (Frost et al., 2014). The status of NL was then tested in the widely used 3xTg mice model of AD. As reported previously (Oddo et al., 2003), we confirmed that these mice display increased level of Aβ deposition before formation of NFT (Figure S1A,B). NL integrity in these studies was assessed using changes in LB1 protein level. Previous studies in Drosophila (Frost et al., 2014, 2016) have shown loss of LB1. Western blotting on hippocampal tissue lysate from two‐ and six‐month‐old 3xTg mouse detected two C‐terminal fragments of LB1: a 46 kDa and a 21 kDa (Figure 1a). We previously showed that the 46 kDa fragment is a product of CASP6 activation (Islam et al., 2019). The reduction in CASP6 and LB1 degradation in 3xTg mouse model was not associated with activation of caspase‐dependent apoptosis as verified with a lack of CASP3 cleavage in these animals (Figure 1a). In this study, we observed that the 21 kDa fragment was also significantly increased in 3xTg mice (*n* = 5–6, *p* < 0.05) (Figure 1a,b) and in hippocampal samples from APP/PS1 mouse, another model of AD that is characterized by increased levels of Aβ (*n* = 4–7, *p* < 0.05) (Figure S1C, D).

The involvement of endo‐lysosomal dysfunction and lysosomal enzymes in AD‐associated neuronal cell death is well documented (Hung & Livesey, 2018; Nixon, 2013, 2017). Accordingly, we detected increased levels of cathepsin B (CTSB) (*n* = 7–8, *p* = 0.059) and cathepsin L (CTSL) (*n* = 7–8, *p* < 0.05) proteins in 3xTg mice (Figure1c,d). The increased protein level was also associated with upregulation of enzymatic activity of CTSL (*n* = 8, *p* < 0.01 and < 0.05) and CTSB (*n* = 5–6, *p* < 0.05) (Figure 1e); however, no change in cathepsin D (CTSD) was noted (Figure 1c,d). Despite changes in lysosomal enzymes, we did not detect any robust changes in autophagy‐related proteins p62, LC3, ATG5, or ATG7 (Figure 1f,g).

### 2.2 LB1 degradation and increased CTSL level are observed in human AD samples

**FIGURE 2:**
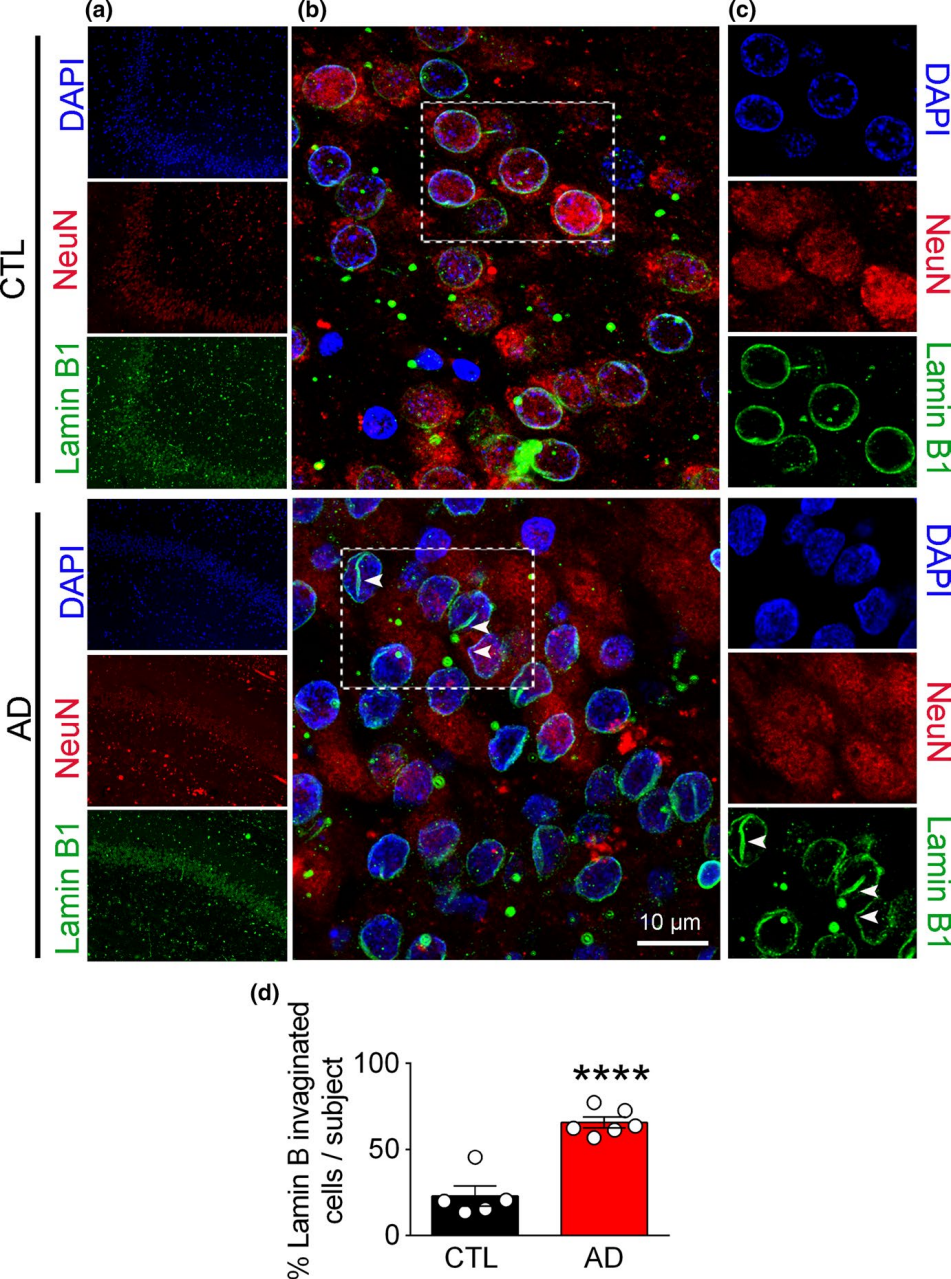
Nuclear lamina damage is detected by invagination and focal loss of Lamin B1 in human AD brain. (a) Confocal microscopic micrographs depicting histological examination of hippocampal and medial temporal cortex from autopsy samples: LB1 (green), NeuN (red), and DAPI (blue). (b and c) Higher magnifications of the selected regions from A are shown (scale bar = 10 µm). (d) Quantification of damaged neuronal nuclei, as identified with their coffee‐bean nuclei immunolabeled with LB1 is shown. Cell counting was performed using ImageJ. A minimum of 109 cells were counted for each sample and the number of invaginated cells were expressed as % of total cells. (CTL = 5 and AD = 6 samples). Data reported as mean ±SEM. **** represents *p* < 0.0001

To examine the relevance of our experimental findings in 3xTg mouse to the pathophysiology of human AD, we assessed the status of NL integrity in postmortem human hippocampal tissue from AD patients and control subjects by using immunohistochemistry and Western blotting. In agreement with a previous report (Frost et al., 2016), we observed that the majority of neuronal nuclei (~65%) in the hippocampus of AD patients displayed NL invagination (*n* = 5–6, *p* < 0.0001) as detected by LB1 labeled coffee‐bean shaped neuronal nuclei (Figure 2a‐d). Additionally, focal loss of LB1 was observed in AD samples (Figure 2b,c). Double staining with NeuN indicated that LB1 damage is a neuronal event as astrocytes identified by glial fibrillary acidic protein (GFAP) did not display any LB1 labeling (Figure S2A).

**FIGURE 3:**
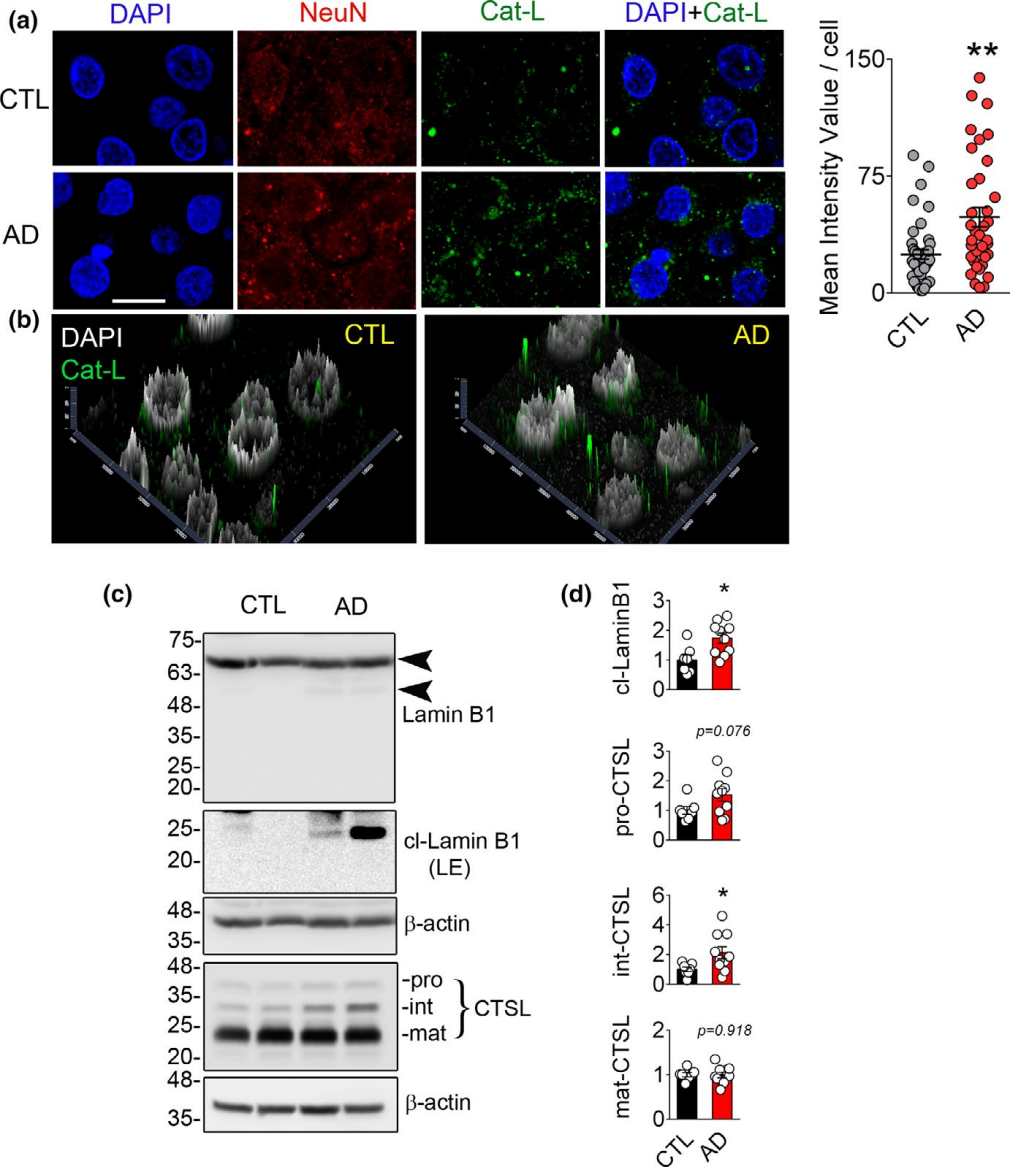
Cathepsin L (CTSL) mediated lamin B1 cleavage is observed in human AD samples. (a) Immunohistochemical labeling of hippocampal and medial temporal sections of human samples for NeuN (red), CTSL (green), and DAPI (blue), scale bar = 10 µm. Mean fluorescence intensity for CTSL was quantified and shown as mean ± SEM, **p < 0.01. (CTL = 5 and AD = 6). (b) Graphs representing distribution and intensity of CTSL in CTL and AD are shown. Higher intensity and closer apposition of CTSL‐positive bodies to the nucleus were observed in AD tissues in comparison with the CTL. (c) Representative Western blotting showing the expression of LB1 and CTSL in human hippocampus samples. (d) Densitometric quantification of the blots is shown as mean ± SEM, CTL (*n* = 7), and AD (*n* = 10), *p < 0.05

Human AD hippocampal sections contained significantly higher levels of neuronal CTSL as detected by quantitative analysis of CTSL signal intensity (*n* = 5–6, *p* < 0.01) in comparison with the controls (Figure 3a). Figure 3b shows the relative intensity and approximate cellular localization of CTSL positive particles; AD samples displayed an increased CTSL signal level and showed a more peri‐nuclear aggregation. Using Western blotting, we further confirmed the increased levels of the 21 kDa cleaved product of LB1 in hippocampal samples from AD patients (*n* = control 7, AD 10, *p* < 0.05) (Figure 3c,d). This was associated with increased levels of intermediate CTSL (*n* = control 7, AD 10, *p* < 0.05) compared to control samples (Figure 3c,d). A ~50 kDa LB1‐positive band was also detected in patient samples; however, it is not clear whether this is mediated by CASP6 activation.

### 2.3 In Vitro Aβ42 toxicity model recapitulates the human neuronal nuclear laminopathy observed in AD

**FIGURE 4:**
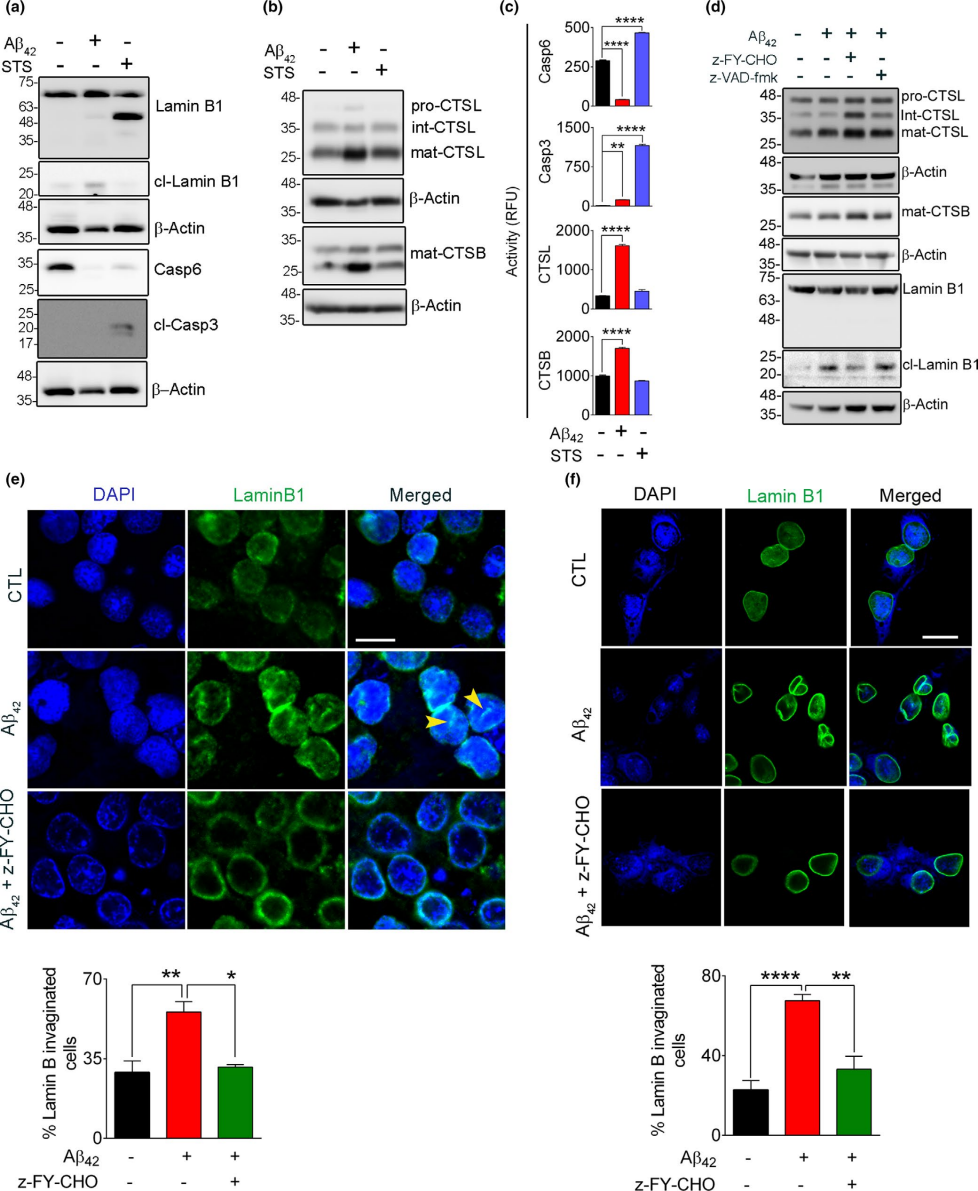
Aβ42‐induced lamin B1 cleavage is independent of apoptosis. To differentiate the contribution of apoptosis and autophagy to lamin B1 cleavage, SH‐SY5Y cells were exposed to Aβ42 (5 µM, 16 h) and STS (0.5 µM, 6 h). (a) Western blots showing the appearance of a lamin B1 46 kDa fragment that was only observed in STS‐treated cells. This was associated with a significant loss of pro‐casp6 protein and presence of cleaved Casp3, indicating the involvement of caspases. The 46 kDa fragment of lamin B1 and pro‐casp6 were not observed in Aβ42‐treated cells and no cleaved Casp3 was detected in these cells. (b) Representative immunoblots examining the changes in lysosomal cathepsin protein level. (c) Enzymatic activity assays for Casp6, Casp3, CTSL, and CTSB indicated the induction of caspases in STS‐treated cells and preferential activation of cathepsins in Aβ42‐treated cells. **p < 0.01 and **** *p* < 0.0001. (one‐way ANOVA/Tukey's post hoc). (d) Western blot confirming the involvement of CTSL in lamin B1 damage in rat primary hippocampal neurons when exposed to Aβ42 (5 µM, 16 h). Overnight pre‐treatment of neurons with CTSL inhibitor alleviated lamin B1 damage, but a pan‐caspase inhibitor z‐VAD‐fmk (20 µM) did not have any effect. (e) Confocal micrographs depicting cultured rat hippocampal neurons labeled for lamin B1 status in Aβ42 toxicity. LB1 invagination was observed in this model but was prevented significantly by CTSL inhibitor. The bar graph represents the percentage of invaginated/nicked neuronal LB1. A minimum of 130 cells/condition were analyzed. (f) The sensitivity of neuronal LB1 damage and NL invagination was also confirmed in mouse primary cortical neurons. This was preventable by pre‐treatment with CTSL inhibitor. An average of 56 cells were examined/experimental group. Results are mean ±SEM, *p < 0.05, **p < 0.01 and ****p < 0.0001, one‐way ANOVA/Tukey's post hoc, scale bar = 10 µm

To further investigate the underpinning mechanisms of LB1 cleavage observed in human AD and 3xTg mouse hippocampal samples, we used an Aβ neuronal toxicity model by exposing SH‐SY5Y neuroblastoma cells to Aβ42. Fibrillogenesis property of Aβ42 was assessed by Thioflavin T assay (Figure S2B). We first tested the fate of LB1 in this model using Western blotting and confirmed the induction of 21 kDa C‐terminal fragment of LB1 (Figure 4a). Cell death in AD has been shown to be mediated by a caspase‐independent apoptosis (Selznick et al., 2000). We therefore compared the status of LB1 in Aβ42 toxicity with Staurosporine (STS) treatment, a well‐known model of caspase‐dependent apoptotic cell death. Western blotting showed (Figure 4a) a robust decrease in pro‐casp6 in both Aβ42 (5uM for 16 hrs) and STS (0.5 µM, 6 h) treated cells. LB1 is a well‐known substrate of CASP6 at cysteine 231, producing a 46 kDa C‐terminal fragment (Ehrnhoefer et al., 2011; Rao et al., 1996); however, the 46 kDa fragment was only observed in STS‐treated cells (Figure 4a). Moreover, cleaved CASP3 was only observed in STS‐treated cells, suggesting that NL damage in Aβ42 toxicity is not associated with induction of apoptosis (Figures 4a,c and S2E). CASP6 activity was decreased significantly in Aβ42‐treated cells (Figure 4c). CTSL and CTSB activity significantly increased only in Aβ42‐treated cells (Figure 4c). An anti‐LB1 antibody that detects the N‐terminal fragments did not show any cleaved products in Aβ42‐treated cells (Figure S3A). Similarly, we were unable to detect any N‐terminal cleaved product in human tissue by Western blotting (data not shown).

Although the appearance of 21 kDa cleaved product of LB1 was observed in Aβ42‐treated cells, there was no apparent reduction in pro‐LB1 compared to control cells (Figures 1a, 3c and 4a). Previous reports (Gerace & Blobel, 1980) have shown that pro‐LB1 is tightly associated with nuclear envelope, and determination of its levels can be affected by cell lysis method. Rigorous cell lysis method, such as those used in our study, results in release of both soluble and membrane‐bound LB1 fraction. We therefore employed a digitonin buffer for gentle lysis of cellular membrane without disruption of nuclear membrane. This approach showed that administration of Aβ42 leads to release of LB1 (21 kDa) in supernatant fraction (sup) that was not present in the control cells (Figure S3B). The pro‐LB1 (67 kDa) was only detected in control cells in the NP‐40‐treated fraction without sonication (ppt), whereas the Aβ42 treated cells contained the 21 kDa fragment (Figure S3B). Despite these obvious differences in sup and ppt fractions, the pellet (sonicated) fraction from control and Aβ42‐treated cells had comparable pro‐LB1 levels, representing the nuclear membrane‐bound LB1 release (Figure S3C). Proteolytic degradation of histone H3, suggesting CTSL activation (Duncan et al., 2008) was also detected in Aβ42‐treated nuclear enriched fraction (Figure S3B). This was further investigated by using purified rhLB1 and rhCTSL. Previous reports have shown that lamins form stable polymeric structures by intra‐ and inter‐molecular bonding (Schirmer & Gerace, 2004) in purified conditions specially if the condition is not denaturing (e.g. presence of 8 M urea). We hypothesized that these global forms were responsible for lack of significant changes in the 67 kDa band density and asked whether increasing the level of rhCTSL can overcome this issue. Increasing amounts of rhCTSL (from 3.12 ng to 200 ng) were incubated with rhLB1. The final concentration of urea in the reaction was 240 mM. Despite successful degradation of rhCTSL and generation of cleaved 21 kDa product at doses as low as 12.5 ng/reaction, the levels of pro‐LB1 (67 kDa) remained relatively unchanged until high doses rhCTSL (100–200 ng/reaction) were used (Figure S3D, E) which was inhibited by CTSL inhibitor (z‐FY‐CHO). Equal high dose of CTSB did not have any effect on rhLB1 (Figure S3D, E).

Similar events were detected in primary hippocampal neurons when exposed to Aβ42. Administration of Aβ42 resulted in appearance of the 21 kDa LB1 fragment (Figures 4d and S3). These events were effectively prevented after pre‐treatment with CTSL inhibitor z‐FY‐CHO. To exclude the involvement of caspases in LB1 damage, cells were treated with z‐VAD‐FMK, an irreversible cell permeable pan‐caspase inhibitor; however, this did not prevent LB1 damage when exposed to Aβ42 (Figure 4d). These data suggested that LB1 is a substrate of CTSL in this model. We then investigated whether treatment of Aβ42 induces LB1 invagination as seen in human AD brain. LB1 invagination/damage was observed in approximately 65% of Aβ42‐treated cells (*n* = 3, *p* < 0.01), but was effectively prevented by z‐FY‐CHO (*n* = 3, *p* < 0.05) (Figure 4e). This finding was further confirmed by administering Aβ42 to primary mouse cortical neurons with or without z‐FY‐CHO. Approximately 70% cells (*n* = 2 experiments, *p* < 0.0001) were observed to have invaginated LB1 after treating with Aβ42, which was prevented by CTSL inhibitor (*p* < 0.01) (Figure 4f).

### 2.4 Aβ42 toxicity increases lysosomal membrane permeability

**FIGURE 5:**
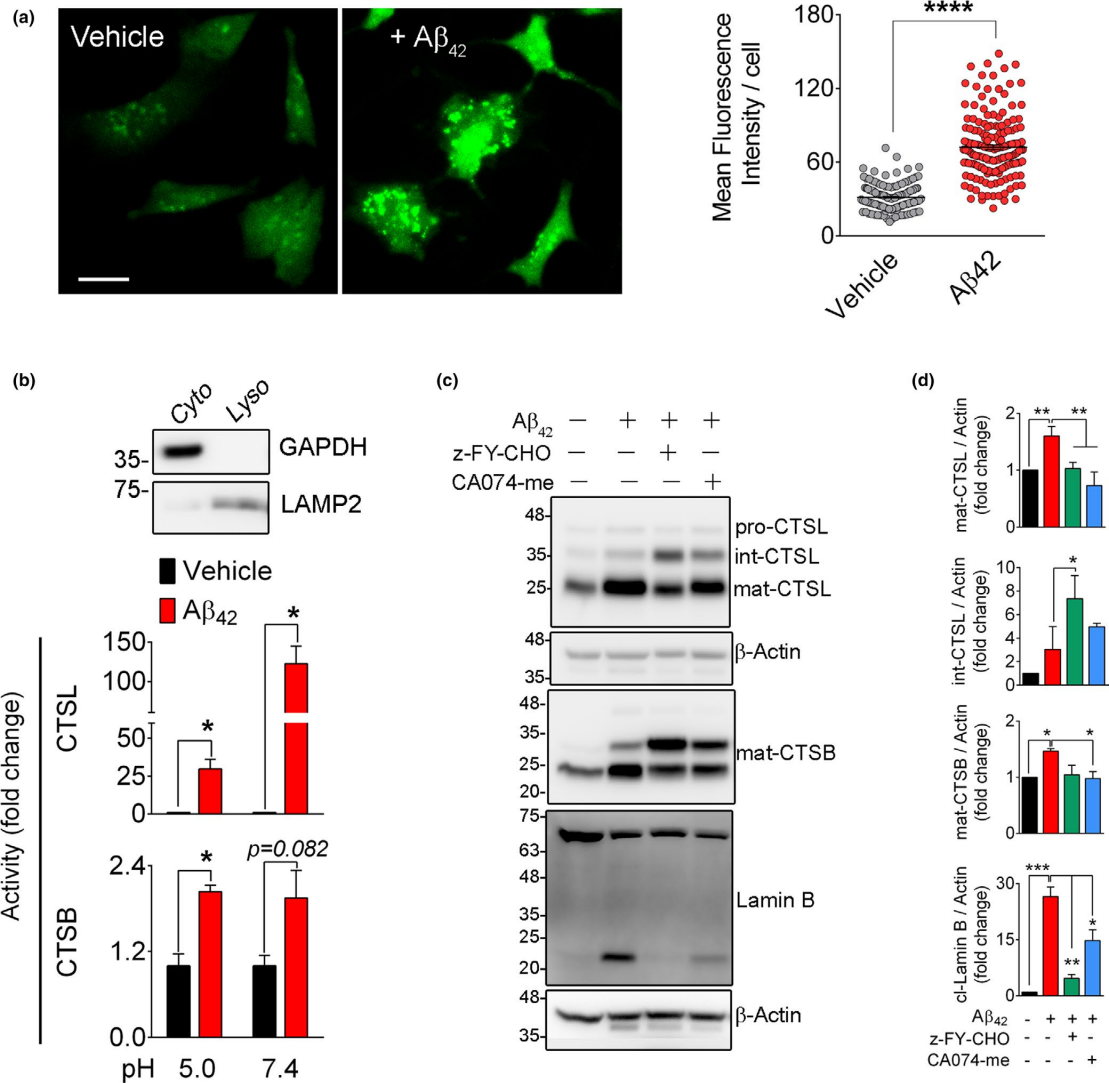
Lamin B1 damage in Aβ42 toxicity is associated with lysosomal membrane permeabilization. (a) Representative acridine orange staining confirmed the induction of LMP in Aβ42‐treated SH‐SY5Y cells. Scale bar = 20 µm. LMP was quantified using green fluorescence intensity/cell. A minimum of 100 cells/condition were examined. ****p < 0.0001. (*t* test) (b) LMP was further assessed by measuring CTSL and CTSB enzymatic activities in cytosolic fractions at pH 5.0 and pH 7.4. Data shown here represents mean ±SEM of *n* = 3 independent experiments. *p < 0.05. The purity of cytosolic fraction was confirmed by a Western blot against β‐actin (cytosol) and LAMP2 (lysosome). (c) Pharmacological inhibition of CTSL prevents Aβ42‐induced lamin B1 cleavage. SH‐SY5Y cells were pre‐treated with indicated inhibitors (20 µM each) overnight. Medium was changed, and cells were exposed to Aβ42 (5 µM) for 16 h. Cathepsin and lamin B were assessed by Western blotting in whole‐cell lysates. (d) Quantification of Western blot results in c, data presented as mean ± SEM, *n* = 3 independent experiments, *p < 0.05, **p < 0.01, and ***p < 0.001, respectively (one‐way ANOVA/Tukey's post hoc). Upregulation of cathepsin B and L coincided with appearance of the 21 kDa lamin B1 product in Aβ42‐treated group. This was robustly prevented by z‐FY‐CHO and partially by CA074‐me

The increase in cathepsin activity in Aβ42‐treated cells (Figure 4c) prompted us to assess the status of lysosomes. LAMP2 (Lysosome‐associated membrane protein 2) levels as a marker of lysosome were increased in coordination with the enhanced expression of cathepsins in Aβ42‐treated cells. Using 3D confocal microscopy and Image J measurement, we noted a significant increase in the size of LAMP2 positive lysosomes in Aβ42‐treated cells (*n* = 2 independent studies, *p* < 0.0001) compared to the vehicle‐treated cells (Figure S4A, B). An increase in lysosomal size has been previously linked to LMP (lysosomal membrane permeabilization) (Boya & Kroemer, 2008). We, therefore, used acridine orange (AO) staining to investigate whether Aβ42 treatment causes LMP. AO is known to accumulate in acidic organelles such as lysosomes, but any loss of lysosomal membrane integrity causes the leakage of AO into the cytosol, where due to change in pH, AO emits green fluorescence (Luo et al., 2018). A robust increase in cytoplasmic green fluorescence after Aβ42 exposure indicated the induction of LMP (Figure 5a) (*n* = 3 independent studies, *p* < 0.0001). We also noted a preferential peri‐nuclear localization of lysosomes as identified by LAMP2 and CTSL staining in Aβ42‐treated cells (Figure S4A,B). To further confirm the induction of LMP and cytoplasmic leakage of lysosomal enzymes, we performed CTSB and CTSL enzymatic activity in cytosolic fraction using their fluorogenic substrates. These reactions were performed at pH 5.0 and pH 7.4 to replicate the lysosomal and cytoplasmic environment, respectively (Duncan et al., 2008). The increase in CTSL activity in Aβ42‐treated cells was several folds higher at pH 7.4 (*n* = 4 independent experiments, *p* < 0.05) than the lysosomal acidic pH 5.0 (Figure 5b). This is in accordance with a previous report where CTSL activity was reported to be maintained at cytosolic pH (Duncan et al., 2008). Elevated CTSB activity was observed at pH 5.0 (*n* = 4, *p* < 0.05) and was not notably affected at pH 7.4 (Figure 5b). The purity of the cytosolic and lysosomal fractions was confirmed by Western blotting (*n* = 4 experiments) (Figure 5b). Distribution of CTSL in cytosolic (sup), nuclear enriched (ppt), and conditioned media was probed by Western blot and an increase in mat‐CTSL was observed in Aβ42‐treated nuclear enriched fraction (Figure S4C). We also found an overall increase in CTSL in Aβ42‐treated conditioned media (Figure S4D) (Noonan et al., 2010).

To examine whether inhibition of these enzymes could prevent LB1 damage, the SH‐SY5Y cells were pre‐treated with cathepsin inhibitors before administration of Aβ42. Pre‐treatment of SH‐SY5Y cells with z‐FY‐CHO, significantly reduced formation of the 21 kDa fragment of LB1 (*n* = 3 experiments, *p* < 0.01) (Figure 5c,d). Quantification of CTSL using Western blotting showed that the mature form of CTSL (~25 kDa) was increased after Aβ42 administration, which was effectively prevented in z‐FY‐CHO pre‐treated cells. This was associated with elevated levels of intermediate isoform of CTSL (34 kDa) (Figure 5c,d). No detectable changes were observed in the level of pro‐CTSL (42 kDa). Although application of CA‐074me, an inhibitor of CTSB, also partially decreased the Aβ42_‐_induced lamin B1 cleavage (*p* < 0.05) (Figure 5c,d), cross‐reactivity of this inhibitor with CTSL has been reported previously (Montaser et al., 2002).

### 2.5 LB1 and CASP6 are substrates of Cathepsin L

**FIGURE 6:**
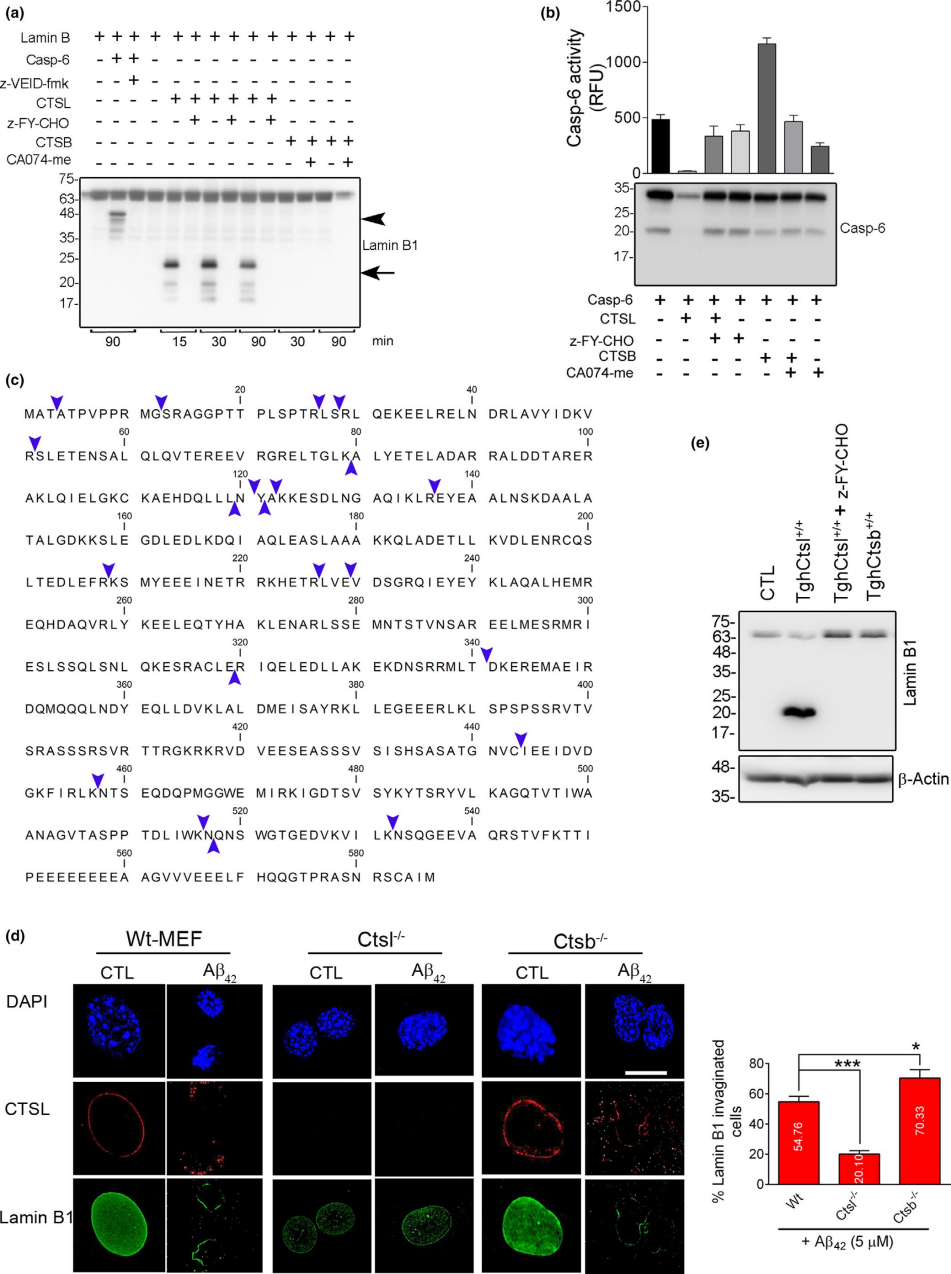
Cathepsin L (CTSL) is the main protease in degradation of lamin B1. (a) Recombinant human (rh) LB1 (1 µg) was incubated with equal amount of (50 ng) rhCasp6, rhCTSL, and rhCTSB at pH 7.4 for the indicated time. Western blot comparing the protease activity of CTSL, CTSB, and CASP6 in processing of LB1, in a cell‐free condition. The 21 kDa fragment of LB1, similar to the one seen in Aβ42 treatment and AD mouse and human samples was only produced by rhCTSL and inhibited by its specific inhibitor. Data are representative of 4 independent experiments. CASP6 produced a ~46 kDa fragment that was inhibited by z‐VEID‐FMK. rhCTSB did not have any effect on rhLB1. (b) Enzymatic activity and Western blotting confirming that CASP6 is degraded by CTSL. rhCasp6 (1 µg) was incubated with rhCTSL and rhCTSB (50 ng each) without/with their specific inhibitors for 1 h at 37°C. Only rhCTSL degraded rhCasp6. These results were further validated using rhCasp6 enzymatic activity using Ac‐VEID‐amc as substrate (bar graph). (c) In vitro reaction mixture of rhLB1 and rhCTSL was subjected to mass spectrometry, and CTSL‐mediated cleavage sites on LB1 were determined by analyzing the small peptides produced in mass spectrometry. The abundance of the peptides was established by considering peptide spectrum match (PSM) 7 or more. The cleavage sites are indicated with blue arrow heads. Peptides are listed in Table S1. (d) Genetic inhibition of CTSL prevents invagination of LB1. Representative 3D confocal microscopy depicting the effect of Aβ42 on LB1 was assessed in WT, *Ctsl^−/−^*, and *Ctsb^−/−^* MEF cells after treatment with 5 µM of Aβ42 for 16 h. Depletion of CTSL^−/−^ effectively prevented LB1 damage. *n* = 3 independent experiments, *p < 0.05, and ***p < 0.001, respectively (one‐way ANOVA/Tukey's post hoc), Scale bar: 10 µm. (e) Western blotting confirmed that overexpression of CTSL (TghCTSL^+/+^) is sufficient to induce LB1 cleavage in normal conditions, however, pre‐treatment with z‐FY‐CHO effectively prevented LB1 damage. WT, *Ctsl^−/−^*, and *Ctsb^−/−^* indicate wilt type, CTSL, and CTSB knock out, respectively

Despite robust decrease in pro‐casp6 level, administration of Aβ42 in SH‐SY5Y and primary neuronal cultures in this study lack of the 46 kDa LB1 fragment negated the involvement of CASP6 in cleavage of LB1. To further confirm these results, we postulated that CASP6 might be degraded in this model, and therefore aimed to examine the interaction of CTSL and CTSB with CASP6 and LB1. Purified recombinant human LB1 (rhLB1) was incubated with rhCASP6, rhCTSL, and rhCTSB and their respective inhibitors at pH 7.4 to recapitulate the potential interaction of these enzymes after lysosomal membrane damage. Our data confirmed two distinct cleavage patterns of rhLB1 for CTSL and CASP6. CTSL‐cleaved rhLB1 with higher efficiency and produced a different cleaved product from CASP6 (Figures 6a and S3D,E). This was completely inhibited by CTSL inhibitor z‐FY‐CHO (Figures 6a and S3D,E). The cleavage pattern of LB1 was similar to that observed after Aβ42 toxicity in SH‐SY5Y (Figure 4a), primary rat hippocampal neurons (Figure 4d), and in 3xTg mouse hippocampal samples (Figure 1a) as well as in human AD brain tissue (Figure 3c). The potential involvement of CTSB in cleavage of LB1 was negated in these experiments (Figure 6a, Figure S3D,E and S5A).

To investigate the lack of CASP6‐mediated LB1 fragment (46 kDa) in Aβ42 toxicity, rhCASP6 was incubated with purified rhCTSL and rhCTSB in the presence and absence of z‐FY‐CHO and CA074‐me, respectively. Our data showed that CTSL, but not CTSB can effectively digest both pro and active forms of CASP6 (Figure 6b). Loss of CASP6 was completely abolished in the presence of the CTSL inhibitor. CTSL mediated degradation of CASP6 was also confirmed by CASP6 enzymatic assay (Figure 6b). To validate these findings in a cellular context, we have incubated whole‐cell extract (SH‐SY5Y) with rhCTSL and rhCTSB. Administration of rhCTSL results in cleavage of LB1, CASP6, and histone H3 in a similar fashion to Aβ42‐treated cells and was successfully prevented by its inhibitor z‐FY‐CHO (Figure S5A,B). The other major cellular cysteine protease, rhCTSB, did not show any effect on LB1, CASP6, and histone H3 (Figure S5A, B).

Taken together, these results suggest that CTSL is the main player in cleaving LB1 in Aβ42‐mediated nuclear damage. The source of the active form of CTSL in NL degradation is not clear. Previous studies (Duncan et al., 2008; Goulet et al., 2004) have reported the presence of a catalytically active form of CTSL in the nucleus; we therefore asked whether activating this nuclear CTSL will produce a similar pattern of lamin B cleavage in isolated nuclei. To test this hypothesis, freshly isolated nuclei from healthy SH‐SY5Y cells were incubated at nuclear pH 7.4 for one hour at 37° C with and without respective cathepsin inhibitors. As expected, CTSL‐mediated cleavage of LB1 was seen, which was inhibited by the CTSL inhibitor (Figure S5C). To identify the specific cleavage sites for CTSL in LB1, we carried out an in vitro reaction of rhCTSL and rhLB1 (pH 7.4) and analyzed the products by mass spectrometry (MS). These data revealed that CTSL cuts most frequently at arginine (R) and lysine (K) on lamin B (Figure 6c). The most favored cleavage site was found to be R136. In addition, there were some instances where CTSL cleaved at a hydrophilic amino acid such as S, E, and T. A list of peptides with their corresponding sequence locations on hLB1 and mass (Da) is shown in Table S1. A decreasing trend in cystatin B protein level (endogenous inhibitor of CTSL) was observed in the AD brain samples (*n* = 3, *p* < 0.01) (Figure S5D,E).

These data collectively indicate that in Aβ42‐mediated cytotoxicity, CTSL acquires a monopoly role in LB1 damage through degradation of CASP6. In line with these observations, significantly decreased level of CASP6 was detected in human AD brain samples compared to CTL (*n* = 3, *p* < 0.05) (Figure S5D,E). Our data indicate a novel role for CTSL in mediation of LB1 damage and induction of NL invagination. However, considering the wide range of CTSL protease activity, the potential contribution of other CTSL substrates in AD pathology cannot be excluded in the present study.

### 2.6 Genetic manipulation of CTSL negatively affects LB1 damage in Aβ42 toxicity

To further examine the role of cathepsins in degradation of LB1 in Aβ42 toxicity model and to confirm the specificity of pharmacologic inhibitors of cathepsins, we employed cultures of MEF cells derived from CTSL and CTSB knockout mice. Cellular phenotype was first confirmed by Western blot and enzymatic activity (Figure S5F,G). This was associated with compensatory upregulation of CTSB protein in *Ctsl* *^−/−^* cells, and increased levels of CTSL in *Ctsb* *^−/−^* Cells (Figure S5F). Similar to SH‐SY5Y cells, treating the wild‐type MEF cells with Aβ42 resulted in increased expression of CTSL which was associated with robust deformation of nucleus and disintegration of LB1 (Figure 6d). These changes were absent in CTSL‐KO MEF cells (Figure 6d). Interestingly, the *Ctsb* *^−/−^* cells, with increased level of CTSL protein had the worst outcome after exposure to Aβ42 (Figure 6d). Overall, these experiments proposed that CTSL‐mediated LB1 degradation in response to Aβ42 treatment is an exclusive phenomenon and can be alleviated by genetic deletion of CTSL. Of particular note, overexpression of human CTSL in transgenic MEF cells (TghCTSL^+/+^) (Figure S5H,I) was sufficient to induce LB1 damage in the absence of any Aβ treatment, as shown by generation of the 21 kDa fragment, which was completely prevented by CTSL inhibitor (Figure 6e). Contrarily, TghCTSB^+/+^ cells did not cause LB1 cleavage (Figure 6e). These data further confirm that LB1 is a specific substrate of CTSL.

### 2.7 LB1 damage affects nuclear architecture and induces histone modification

**FIGURE 7:**
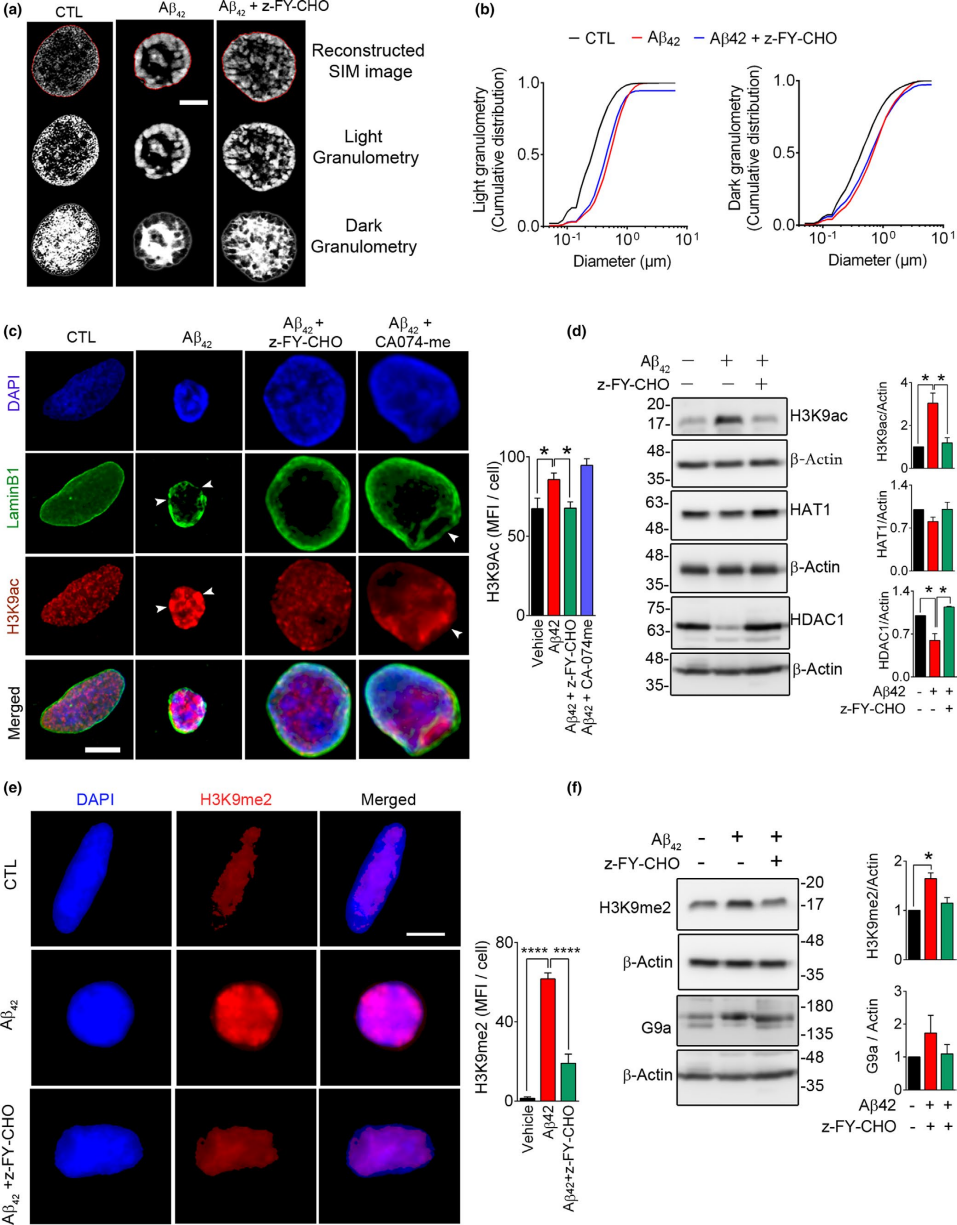
Lamin B1 damage in Aβ42 toxicity affects nuclear architecture and chromatin remodeling. To examine the effect of LB1 invagination observed in Aβ42 toxicity, we exposed the SH‐SY5Y cells to Aβ42 (5 µM, 16 h), with/without pre‐treatment with CTSL inhibitor (z‐FY‐CHO, 20 µM). (a) Micrographs representing 3D‐Struture Illumination Microscopy (3D‐SIM) of DAPI‐stained cells. (Scale bar = 1 µm) (b) Granulometry analysis of images in a is shown by graph (cumulative distribution vs size in µm). A minimum of 39 cells/condition were analyzed. Data are representative of two independent experiments and reported as mean ± SEM. Statistical analysis (one‐way ANOVA/Tukey's post hoc) showed that administration of Aβ42 significantly increased DNA compaction, as shown by light granulometry: (*p* = 1.1744E‐138) that was prevented in cells pre‐treated with z‐FY‐CHO (*p* = 0.700E‐04). DNA‐free space, assessed by dark granulometry, was also increased after Aβ42 administration (CTL vs. Aβ42, *p* = 5.7328E‐17), although it did not fully recover after inhibition of CTSL (Aβ42 + z‐FY‐CHO: *p* = 0.5810). (c) Representative 3D confocal microscopy depicting epigenetic changes (H3K9ac) associated with lamin B1 damage. While inhibition of CTSL (z‐FY‐CHO, 20 µM) robustly alleviated these changes, inhibition of CTSB (CA074‐me, 20 µM) was not equally effective. Quantitative analysis of H3K9ac mean fluorescence intensity (MFI)/cells (*n *≥ 46 cells/treatment group) is shown as a bar graph. Data are representative of three independent experiments and reported as mean ± SEM, *p < 0.05 (one‐way ANOVA/Tukey's post hoc), Scale bar = 10 µm. (d) To gain a mechanistic view of molecular changes in acetylation, Western blotting was performed, showing that the increase in H3K9ac was due to decreased HDAC1 levels in Aβ42 treated cells and was normalized by inhibition of CTSL. *n* = 2 independent experiments, data are mean ± SEM. *p < 0.05 (one‐way ANOVA/Tukey's post hoc). (e) Confocal micrographs depicting changes in H3K9me2 in this experimental model. Mean fluorescence intensity for H3K9me2 was analyzed (*n* ≥ 41 cells/conditions) and reported as mean ±SEM. ****p < 0.0001 (one‐way ANOVA/Tukey's post hoc), Scale bar: 10 µm. (f) H3K9me2 and G9a were probed for Western blotting after treating SH‐SY5Y cells as stated in e. Quantitative bar graphs of the indicated protein levels. Data reported as mean ± SEM. *p < 0.05 (one‐way ANOVA/Tukey's post hoc)

A robust change in nuclear size and DNA density was noted in cells exposed to Aβ42. We therefore employed 3D‐SIM super‐resolution microscopy to quantitatively measure changes in DNA organization. This was achieved by granulometry, a method that is used for measuring DNA compaction and DNA‐free/poor space in nuclei (Righolt et al., 2014). We observed that Aβ42 treatment caused robust changes in compaction of nuclear chromatin (*n* = 2 experiments, *p* = 1.1744E‐138) and significant increase in DNA‐free space (*p* = 5.7328E‐17) in SH‐SY5Y cells (Figure 7a,b). Pre‐treatment of these cells with z‐FY‐CHO, significantly prevented DNA compaction (light granulometry: *p* = 0.700E‐04), although changes in DNA‐free space were not statistically significant (dark granulometry: *p* = 0.5810) (Figure 7a,b).

The involvement of NL in regulation of gene expression has been attributed to its physical contact with lamin‐associated domains (LAD) and induction of epigenetic changes in nuclear chromatin. We therefore asked whether changes in CTSL‐LB1 axis are involved in induction of such changes. 3D confocal microscopy in immunolabeled SH‐SY5Y cells was used to examine the effect of Aβ42 treatment on the extent of histone modification. Using antibodies to H3K9ac and H3K9me2 as indicators of acetylation and methylation, representing epigenetic changes, we detected significant overall increase in histone acetylation (*n* = 3 experiments, *p* < 0.05). The intensity of H3K9ac staining was specifically higher in areas where LB1 integrity was compromised (Figure 7c, white arrow heads). Pre‐treatment of cells with CTSL inhibitors prevented the overall increase in H3K9 acetylation (*p*<0.05) (Figure 7c). Aβ42‐induced hyperacetylation of histone H3 at K9 was further verified by Western blot analysis in cell extracts and was ameliorated after CTSL inhibition (*n* = 2, *p* < 0.05) (Figure 7d). We then focused to better understand the underlying mechanisms involved in regulation of H3 acetylation. While histone acetyltransferase‐1 (HAT1) did not change after Aβ42 treatment (*n* = 2), histone deacetylase 1 (HDAC1) was robustly decreased (*p* < 0.05) in these cells but returned to normal levels in the presence of CTSL inhibitor (*p* < 0.05) (Figure 7d). A significant increase in chromatin methylation was detected by assessment of H3K9me2 signal intensity after administration of Aβ42 in SH‐SY5Y (*n* = 3, *p* < 0.0001), which was effectively lowered by CTSL inhibition (*p* < 0.0001) (Figure 7e). These results were further confirmed by Western blotting in Aβ42 treated cell extracts (*n* = 2, *p* < 0.05) (Figure 7f). To check the applicability of these findings in an animal model of AD, the protein levels of H3K9ac, HeK9me2, HAT1, and HDAC1 were assessed in the hippocampus of 3xTg mice at 2‐ and 6‐month age. Although there was no detectable difference in H3K9ac levels at 2 months, H3K9ac levels were significantly (*p* < 0.01) upregulated at 6 months (Figure S6A,B). Conversely, the 2‐month‐old 3xTg mice displayed significantly higher protein levels of HDAC1 (*n* = 3, *p* < 0.01), HAT1 (*n* = 3, *p* < 0.05), and H3K9me2 (*n* = 4–5, *p* < 0.05) than the wild‐type controls. These differences disappeared in 6‐month‐old mice (Figure S6A,B). The findings were further validated using human AD brain samples. Previous reports have shown an increase in acetylation of histone H3 at lysine 9 residues (K9) in AD (Klein et al., 2019). We also found an overall increasing tendency in H3K9Ac (*n* = 3, *p* = 0.238) that coincided with a significant increase (*n* = 3, *p* < 0.05) in HAT1 in AD brain tissues (Figure S6C,D). Similarly, treatment of primary mouse cortical neurons with Aβ42‐induced LB1 invagination and increased acetylation of histone at K9 (*n* = 2, *p* < 0.0001) which was ameliorated by CTSL inhibitor (*n* = 2, *p* < 0.01) (Figure S6E).

## 3 DISCUSSION

In the present study, we have identified CTSL as an important player in regulation of NL integrity in an in vitro model of Aβ toxicity. This was associated with significant structural changes that altered chromatin organization and caused histone modifications. These changes were substantially alleviated after pharmacologic and genetic downregulation of CTSL. Further support for CTSL involvement in nuclear changes was obtained in the 3xTg mouse model of AD and in human postmortem hippocampal tissue. Neuronal NL damage is a newly identified frontier in AD pathology and our discovery unraveling the key role of CTSL in degradation of LB1 is an important finding that fills the gap in the field and may provide important information for developing new therapeutic strategies.

### 3.1 Nuclear lamina degradation is a prominent feature of Alzheimer's disease pathophysiology

The importance of NL integrity in cell's functional and structural health is mediated by its anchoring role for nuclear chromatin, providing a functional compartmentalization for gene expression. The genome is divided into smaller sub‐domains A and B (Dixon et al., 2015). Genes associated with compartment A are considered to be active and mostly euchromatic, whereas, B compartment associated genes are inactive (Lieberman‐Aiden et al., 2009) and heterochromatic. A tightly regulated dynamic relationship exists between the domains, that is, a gene can move between the compartments (Dixon et al., 2015), and this is controlled by the association of these compartments with other functional parts such as NL (Reddy et al., 2008; Zullo et al., 2012). Nuclear LB1 is related to compartment B and restricts the accessibility/transcription of LB1‐tethered genes (Reddy et al., 2008). It is therefore expected that any disruptions of LB1 affect cellular function. LB1 physical interaction with Oct1 transcription factor is vital for neuronal survival under oxidative stress (Malhas et al., 2009), and hence, genetic deletion of LB1 or Oct1 has been shown to result in dysregulation of various systems including those relevant to oxidative stress and aging (Malhas et al., 2009). This regulatory role of LB1 has also been shown in models of neurodegeneration (Frost et al., 2016).

The available literature indicates the involvement of NL damage in pathophysiology of AD in a wide range of in vitro and in vivo animal models as well as in postmortem brain tissue from AD patients. Earlier genetic and pharmacologic models showed that targeting cellular antioxidants result in NL damage and neurodegeneration (Dias‐Santagata et al., 2007; Frost et al., 2014). These studies proposed that NL damage is a consequence of Tau hyperphosphorylation (Frost et al., 2014) and leads to aberrant gene expression (Cornelison et al., 2019; Sun et al., 2018). Research from our group further proposes the involvement of oxidative stress, as mediated by inhibition of thioredoxin system and activation of caspase‐6 (Islam et al., 2019). Using 3xTg mice in which Aβ deposition occurs before NFT formation, the present study suggests that LB1 damage is an early event that can be detected in hippocampus as early as 2 months of age. Aβ deposition and NFT formation in this model are reported to occur between 6 and 12 months of age (Oddo et al., 2003). We further used an in vitro model of Aβ42 toxicity and showed that LB1 damage can also be induced by Aβ42 toxicity.

Collectively, the available literature from different models of AD disease including Tau toxicity, genetic and pharmacologic inhibition of Trx system, and our current observation with Aβ42 model, indicate that LB1 degradation is a common event in these models, however, whether this is the cause or consequence of these changes remain unknown.

### 3.2 Cysteine cathepsins and NL damage in AD

In these studies, we showed LB1 damage coincided with increased lysosomal size and membrane permeability and was associated with elevated CTSL protein level and enzymatic activity in Aβ42 toxicity model, hippocampal lysate from 3xTg mouse and in human brain tissue from AD patients. Using pharmacological inhibitor, we showed that CTSL inhibition can successfully inhibit NL damage. Proof of principle data from *Ctsl^−/−^* MEF cells confirmed the specificity of CTSL while genetic depletion (*Ctsb^−/−^*) or overexpression (Tg*Ctsb^+/+^*), did not have any major effect on LB1 degradation. In fact, the *Ctsb^−/−^* cells displayed the worst degradation of LB1 when exposed to Aβ42. There are conflicting reports on the role of CTSB in Aβ toxicity in AD, identifying CTSB as a protective protease by digesting Aβ and reducing its accumulation in the neuronal cells (Mueller‐Steiner et al., 2006; Perlenfein & Murphy, 2017; Wang et al., 2012; Zhou et al., 2020). Supporting this notion, overexpressing CTSB’s endogenous inhibitor cystatin C had been proposed to initiate the onset of AD (Wang et al., 2012). On the contrary, overexpressing cystatin C was also shown to reduce Aβ load in AD mice (Mi et al., 2007), suggesting a harmful effect for CTSB in Aβ metabolism. In our study, application of Aβ42 in cultures of MEF cells lacking CTSB, exacerbated nuclear morphology, although this may be the result of impaired Aβ clearance due to the lack of CTSB, but it also can be a reflection of compensatory increased expression of CTSL in these cells. Overall, our studies showed that CTSL and not CTSB is involved in maintaining nuclear integrity in these experimental conditions.

Upregulation of cathepsin L activity in these experiments was associated with degradation of CASP6 and inhibition of its activity, perhaps proposing an anti‐apoptotic activity, for CTSL. This was supported by lack of Casp3 activation in Aβ42 toxicity model and in 3xTg mouse. A similar anti‐apoptotic or pro‐autophagy role for CTSL has been previously reported through its capacity to degrade CTSD, which in turn is an activator of CASP3‐mediated apoptosis (Zheng et al., 2008). Our results propose that CTSL activation delays the onset of apoptosis by inhibiting caspases, and therefore, are in agreement with a previous report showing that classical apoptosis was only induced in late stages of AD pathology (Guglielmotto et al., 2014).

### 3.3 Cathepsin L involvement in nuclear reorganization

The importance of CTSL has been shown in a variety of biological processes from cell division to cell death. We provide evidence that a catalytic form of CTSL is activated in the nuclear compartment and contributes to NL damage in Aβ42‐mediated toxicity. A similar observation has been previously reported involving CTSL regulation of cell proliferation through processing of CDP/Cux transcription factor in NIH3T3 (Goulet et al., 2004). The authors showed that this catalytic form of CTSL is shorter, lacking a signal peptide and is targeted to nuclear compartment. Accordingly, inhibition of CTSL is shown to disrupt cell proliferation in cancer cells (Tamhane et al., 2016). Another evidence for nuclear activity of CTSL has been shown in differentiation of mouse embryonic stem cells that is regulated by cleavage of histone H3 (Duncan et al., 2008). Activation of CTSL has been linked to degradation of DNA repair machinery, for example, 53BP1, pRb, and p107 (Das et al., 2013; Grotsky et al., 2013).

In our study, we provide the first evidence that CTSL can cause structural and functional changes to NL by cleaving LB1. We have shown that degradation of NL in Aβ42 toxicity results in overall shrinkage of the nucleus, increased compaction of DNA, and DNA‐free space in the nucleus. This is in agreement with a previous study showing increased chromatin density in Aβ toxicity model (Estus et al., 1997). Immunostaining data revealed a robust increase in H3K9ac in regions with low LB1 signals, indicating that LB1 level negatively regulates acetylation. This is in agreement with a recent report showing extensive acetylation in tauopathy and AD (Klein et al., 2019). Whether the cleaved LB1 fragments play direct role in induction of these changes remains to be further examined. Additionally, hypermethylation of H3 is another event in AD that has been linked to changes in gene expressions (Frost et al., 2014, 2016). We also observed an overall increase in H3 methylation at K9 in response to Aβ42 treatment in SH‐SY5Y cells, which was further confirmed in 3xTg mice. However, as CTSL has a wide range of nuclear substrates (discussed above), the potential contribution of other CTSL substrates in nuclear events cannot be negated. This area deserves further research for identification of downstream players after CTSL activation and may lead to discovery of more effective therapeutic target.

### 3.4 Limitations of the study

In this study, we have used undifferentiated SH‐SY5Y neuroblastoma, primary cortical, and hippocampal neurons from E18 mouse embryo to study the molecular events associated with Aβ42 toxicity. While these models have been extensively used for shaping our current knowledge of neurodegeneration process in AD, the chronic nature of neurodegeneration cannot be appropriately examined under these in vitro conditions and must be complemented by animal models and available human tissue. Accordingly, using the widespread 3xTg mouse model of AD that display evidence of tauopathy and amyloid beta toxicity before the onset of cognitive deficits, we showed the relevance of these finding in human brain samples from AD patients and controls. However, one must consider the inevitable limitations of accessing human samples including the variance in postmortem tissue retrieval.

### 3.5 Conclusion

Collectively, our findings in the current study are suggestive of a central role for CTSL in induction of NL damage in models of AD. We have shown that upregulation of CTSL is associated with LB1 digestion and induction of NL invagination. Robust chromatin reorganization and histone modifications occurred in the model of Aβ42 toxicity, which was alleviated by inhibition of CTSL. We are proposing new enzyme‐substrate (CTSL‐LB1) system for the first time with experimental evidence. The applicability of these findings in targeting CTSL as a therapeutic approach in AD animal models will be an exciting future research direction. This will require novel methods for in vivo modulation of the neuronal CTSL/LB1 axis.

## 4 MATERIALS AND METHODS

### 4.1 Reagents and antibodies

Reagents and antibodies used for the study are listed in Tables S2a and S2b, respectively.

### 4.2 Animals

3xTg mice (a gift from Dr. Mark Mattson, National Institutes of Health, Baltimore, Maryland), were maintained on a C57BL/6 background for eight generations. Only homozygous mice (four males and four females for each time point) for the transgene and their age‐matched wild‐type controls were used for the study. The 3xTg mice express three transgene (*APPswe*, *PS1M146V*, and *Tau P301L*) and show evidence of amyloid plaque and NFT formation, with early signs of mild cognitive impairment at six months of age (Stover et al., 2015). The deposition of amyloid beta plaques occurs before formation of NFT in this model (Oddo et al., 2003). We additionally used APP/PS1 mice generated from cross‐breeding of a single transgenic mice expressing human APPK670N/M671L with mice expressing human PS1M146L (Y. Wang et al., 2019). All animal use was carried out according to standard protocols approved by the University of Manitoba Animal Care Committee in accordance with the Canadian Council on Animal Care guidelines and policies. Animals were euthanized at indicated age and their left and right hippocampi tissue were collected. For protein analysis, the hippocampal tissue was lysed in a lysis buffer (50 mM Tris‐HCl pH 8.0, 150 mM NaCl, 5 mM EDTA, 1% v/v NP‐40) by manual homogenization followed by sonication (10s X 3) on ice. Samples were then centrifuged for 30 min at 16,200 *g* at 4°C. The supernatant was used immediately for measurement of cathepsin L (CTSL) and cathepsin B (CTSB) enzymatic activity. The remaining supernatant was treated with protease and phosphatase inhibitors for Western blot sample preparation according to our routine procedures (Islam et al., 2019).

### 4.3 Lysosome size analysis

3D confocal microscopy images were converted to 8‐bit images, and the size of the lysosomes were determined using Image J software. “Analyze particles” tool was used with a set parameter of Threshold = Auto (Triangle method); Size = 0.1–1.5 µm^2^, Circularity = 0.05–1.00, Scale = 9.76 pixel/µm.

### 4.4 Statistical analysis

We used GraphPad Prism version 6.00 for Windows. For comparing two experimental groups, Student's *t* test was used. One‐way ANOVA followed by Tukey's post hoc test was performed to compare more than two experimental groups. Sample size and significance level have been indicated in the figures and legends.

## CONFLICT OF INTEREST

All the authors declare no competing of interests.

## AUTHOR CONTRIBUTIONS

MII conceptualized, designed and performed the experiments, analyzed the experimental data, and wrote the first draft of the manuscript. NP performed data acquisition and interpretation, and manuscript review and editing. TS took part in counting the 3D images for invagination. FC trained MII for 3D confocal microscopy and interpret the data. SM provided the facility for 3D confocal microscopy, review and edit the manuscript. BA provided the mouse hippocampal tissue, reviewed and edited the manuscript. MDB provided the human samples for IHC and WB, interpreted the data, reviewed and edited the manuscript. JFW provided the hippocampal samples for APP/PS1 mouse. MGS and RY provided samples and Western blot data for human hippocampal samples. ISP provided Aβ42, reviewed and edited the manuscript. EE conceptualized, designed and supervised the experiments, data acquisition, and interpretation of results, edited the final version of manuscript, and acquired funding for the project. All authors read and approved the final manuscript.

## Supporting information

Figure S1

Figure S2

Figure S3

Figure S4

Figure S5

Figure S6

Tables S1–S3

## Data Availability

The data that support the findings of this study are available from the corresponding author upon reasonable request.

## References

[acel13531-bib-0001] Arranz, A. M. , & De Strooper, B. (2019). The role of astroglia in Alzheimer's disease: Pathophysiology and clinical implications. The Lancet Neurology, 18(4), 406–414. 10.1016/S1474-4422(18)30490-3 30795987

[acel13531-bib-0002] Bertero, A. , Fields, P. A. , Smith, A. S. T. , Leonard, A. , Beussman, K. , Sniadecki, N. J. , Kim, D.‐H. , Tse, H.‐F. , Pabon, L. , Shendure, J. , Noble, W. S. , & Murry, C. E. (2019). Chromatin compartment dynamics in a haploinsufficient model of cardiac laminopathy. Journal of Cell Biology, 218(9), 2919–2944. 10.1083/jcb.201902117 PMC6719452 31395619

[acel13531-bib-0003] Boya, P. , & Kroemer, G. (2008). Lysosomal membrane permeabilization in cell death. Oncogene, 27(50), 6434–6451. 10.1038/onc.2008.310 18955971

[acel13531-bib-0004] Broers, J. L. , Ramaekers, F. C. , Bonne, G. , Yaou, R. B. , & Hutchison, C. J. (2006). Nuclear lamins: Laminopathies and their role in premature ageing. Physiological Reviews, 86(3), 967–1008. 10.1152/physrev.00047.2005 16816143

[acel13531-bib-0005] Butin‐Israeli, V. , Adam, S. A. , Jain, N. , Otte, G. L. , Neems, D. , Wiesmüller, L. , Berger, S. L. , & Goldman, R. D. (2015). Role of lamin b1 in chromatin instability. Molecular and Cellular Biology, 35(5), 884–898. 10.1128/MCB.01145-14 25535332 PMC4323489

[acel13531-bib-0006] Chang, K.‐H. , Multani, P. S. , Sun, K.‐H. , Vincent, F. , de Pablo, Y. , Ghosh, S. , Gupta, R. , Lee, H.‐P. , Lee, H.‐G. , Smith, M. A. , & Shah, K. (2011). Nuclear envelope dispersion triggered by deregulated Cdk5 precedes neuronal death. Molecular Biology of the Cell, 22(9), 1452–1462. 10.1091/mbc.E10-07-0654 21389115 PMC3084668

[acel13531-bib-0007] Chen, N. Y. , Yang, Y. E. , Weston, T. A. , Belling, J. N. , Heizer, P. , Tu, Y. , Kim, P. , Edillo, L. , Jonas, S. J. , Weiss, P. S. , Fong, L. G. , & Young, S. G. (2019). An absence of lamin B1 in migrating neurons causes nuclear membrane ruptures and cell death. Proceedings of the National Academy of Sciences of the United States of America, 116(51), 25870–25879. 10.1073/pnas.1917225116 31796586 PMC6926041

[acel13531-bib-0008] Coffinier, C. , Chang, S. Y. , Nobumori, C. , Tu, Y. , Farber, E. A. , Toth, J. I. , Fong, L. G. , & Young, S. G. (2010). Abnormal development of the cerebral cortex and cerebellum in the setting of lamin B2 deficiency. Proceedings of the National Academy of Sciences of the United States of America, 107(11), 5076–5081. 10.1073/pnas.0908790107 20145110 PMC2841930

[acel13531-bib-0009] Cornelison, G. L. , Levy, S. A. , Jenson, T. , & Frost, B. (2019). Tau‐induced nuclear envelope invagination causes a toxic accumulation of mRNA in Drosophila. Aging Cell, 18(1), e12847. 10.1111/acel.12847 30411463 PMC6351838

[acel13531-bib-0010] Das, A. , Grotsky, D. A. , Neumann, M. A. , Kreienkamp, R. , Gonzalez‐Suarez, I. , Redwood, A. B. , & Gonzalo, S. (2013). Lamin A Deltaexon9 mutation leads to telomere and chromatin defects but not genomic instability. Nucleus, 4(5), 410–419. 10.4161/nucl.26873 24153156 PMC3899131

[acel13531-bib-0011] Dias‐Santagata, D. , Fulga, T. A. , Duttaroy, A. , & Feany, M. B. (2007). Oxidative stress mediates tau‐induced neurodegeneration in Drosophila. Journal of Clinical Investigation, 117(1), 236–245. 10.1172/JCI28769 PMC1697799 17173140

[acel13531-bib-0012] Dixon, J. R. , Jung, I. , Selvaraj, S. , Shen, Y. , Antosiewicz‐Bourget, J. E. , Lee, A. Y. , Ye, Z. , Kim, A. , Rajagopal, N. , Xie, W. , Diao, Y. , Liang, J. , Zhao, H. , Lobanenkov, V. V. , Ecker, J. R. , Thomson, J. A. , & Ren, B. (2015). Chromatin architecture reorganization during stem cell differentiation. Nature, 518(7539), 331–336. 10.1038/nature14222 25693564 PMC4515363

[acel13531-bib-0013] Dou, Z. , Xu, C. , Donahue, G. , Shimi, T. , Pan, J.‐A. , Zhu, J. , Ivanov, A. , Capell, B. C. , Drake, A. M. , Shah, P. P. , Catanzaro, J. M. , Daniel Ricketts, M. , Lamark, T. , Adam, S. A. , Marmorstein, R. , Zong, W.‐X. , Johansen, T. , Goldman, R. D. , Adams, P. D. , & Berger, S. L. (2015). Autophagy mediates degradation of nuclear lamina. Nature, 527(7576), 105–109. 10.1038/nature15548 26524528 PMC4824414

[acel13531-bib-0014] Duncan, E. M. , Muratore‐Schroeder, T. L. , Cook, R. G. , Garcia, B. A. , Shabanowitz, J. , Hunt, D. F. , & Allis, C. D. (2008). Cathepsin L proteolytically processes histone H3 during mouse embryonic stem cell differentiation. Cell, 135(2), 284–294. 10.1016/j.cell.2008.09.055 18957203 PMC2579750

[acel13531-bib-0015] Duyckaerts, C. , Delatour, B. , & Potier, M. C. (2009). Classification and basic pathology of Alzheimer disease. Acta Neuropathologica, 118(1), 5–36. 10.1007/s00401-009-0532-1 19381658

[acel13531-bib-0016] Ehrnhoefer, D. E. , Skotte, N. H. , Savill, J. , Nguyen, Y. T. N. , Ladha, S. , Cao, L.‐P. , Dullaghan, E. , & Hayden, M. R. (2011). A quantitative method for the specific assessment of caspase‐6 activity in cell culture. PLoS One, 6(11), e27680. 10.1371/journal.pone.0027680 22140457 PMC3226564

[acel13531-bib-0017] Estus, S. , Tucker, H. M. , van Rooyen, C. , Wright, S. , Brigham, E. F. , Wogulis, M. , & Rydel, R. E. (1997). Aggregated amyloid‐beta protein induces cortical neuronal apoptosis and concomitant "apoptotic" pattern of gene induction. Journal of Neuroscience, 17(20), 7736–7745. 9315895 10.1523/JNEUROSCI.17-20-07736.1997 PMC6793913

[acel13531-bib-0018] Frost, B. (2016). Alzheimer's disease: An acquired neurodegenerative laminopathy. Nucleus, 7(3), 275–283. 10.1080/19491034.2016.1183859 27167528 PMC4991240

[acel13531-bib-0019] Frost, B. , Bardai, F. H. , & Feany, M. B. (2016). Lamin dysfunction mediates neurodegeneration in tauopathies. Current Biology, 26(1), 129–136. 10.1016/j.cub.2015.11.039 26725200 PMC4713335

[acel13531-bib-0020] Frost, B. , Hemberg, M. , Lewis, J. , & Feany, M. B. (2014). Tau promotes neurodegeneration through global chromatin relaxation. Nature Neuroscience, 17(3), 357–366. 10.1038/nn.3639 24464041 PMC4012297

[acel13531-bib-0021] Gerace, L. , & Blobel, G. (1980). The nuclear envelope lamina is reversibly depolymerized during mitosis. Cell, 19(1), 277–287. 10.1016/0092-8674(80)90409-2 7357605

[acel13531-bib-0022] Goulet, B. , Baruch, A. , Moon, N.‐S. , Poirier, M. , Sansregret, L. L. , Erickson, A. , Bogyo, M. , & Nepveu, A. (2004). A cathepsin L isoform that is devoid of a signal peptide localizes to the nucleus in S phase and processes the CDP/Cux transcription factor. Molecular Cell, 14(2), 207–219. 10.1016/s1097-2765(04)00209-6 15099520

[acel13531-bib-0023] Grotsky, D. A. , Gonzalez‐Suarez, I. , Novell, A. , Neumann, M. A. , Yaddanapudi, S. C. , Croke, M. , Martinez‐Alonso, M. , Redwood, A. B. , Ortega‐Martinez, S. , Feng, Z. , Lerma, E. , Ramon y Cajal, T. , Zhang, J. , Matias‐Guiu, X. , Dusso, A. , & Gonzalo, S. (2013). BRCA1 loss activates cathepsin L‐mediated degradation of 53BP1 in breast cancer cells. Journal of Cell Biology, 200(2), 187–202. 10.1083/jcb.201204053 PMC3549967 23337117

[acel13531-bib-0024] Guglielmotto, M. , Monteleone, D. , Piras, A. , Valsecchi, V. , Tropiano, M. , Ariano, S. , & Tamagno, E. (2014). Abeta1‐42 monomers or oligomers have different effects on autophagy and apoptosis. Autophagy, 10(10), 1827–1843. 10.4161/auto.30001 25136804 PMC4198366

[acel13531-bib-0025] Halawani, D. , Tessier, S. , Anzellotti, D. , Bennett, D. A. , Latterich, M. , & LeBlanc, A. C. (2010). Identification of Caspase‐6‐mediated processing of the valosin containing protein (p97) in Alzheimer's disease: A novel link to dysfunction in ubiquitin proteasome system‐mediated protein degradation. Journal of Neuroscience, 30(17), 6132–6142. 10.1523/JNEUROSCI.5874-09.2010 20427671 PMC3187624

[acel13531-bib-0026] Hardy, J. A. , & Higgins, G. A. (1992). Alzheimer's disease: The amyloid cascade hypothesis. Science, 256(5054), 184–185. 10.1126/science.1566067 1566067

[acel13531-bib-0027] Hohn, A. , Tramutola, A. , & Cascella, R. (2020). Proteostasis failure in neurodegenerative diseases: Focus on oxidative stress. Oxidative Medicine and Cellular Longevity, 2020, 5497046. 10.1155/2020/5497046 32308803 PMC7140146

[acel13531-bib-0028] Hung, C. O. Y. , & Livesey, F. J. (2018). Altered gamma‐Secretase processing of APP disrupts lysosome and autophagosome function in monogenic Alzheimer's disease. Cell Reports, 25(13), 3647–3660 e3642. 10.1016/j.celrep.2018.11.095 30590039 PMC6315085

[acel13531-bib-0029] Islam, M. I. , Nagakannan, P. , Ogungbola, O. , Djordjevic, J. , Albensi, B. C. , & Eftekharpour, E. (2019). Thioredoxin system as a gatekeeper in caspase‐6 activation and nuclear lamina integrity: Implications for Alzheimer's disease. Free Radical Biology and Medicine, 134, 567–580. 10.1016/j.freeradbiomed.2019.02.010 30769159

[acel13531-bib-0030] Jack, C. R. , Bennett, D. A. , Blennow, K. , Carrillo, M. C. , Dunn, B. , Haeberlein, S. B. , Holtzman, D. M. , Jagust, W. , Jessen, F. , Karlawish, J. , Liu, E. , Molinuevo, J. L. , Montine, T. , Phelps, C. , Rankin, K. P. , Rowe, C. C. , Scheltens, P. , Siemers, E. , Snyder, H. M. , … Silverberg, N. (2018). NIA‐AA research framework: Toward a biological definition of Alzheimer's disease. Alzheimer's & Dementia: the Journal of the Alzheimer's Association, 14(4), 535–562. 10.1016/j.jalz.2018.02.018 PMC5958625 29653606

[acel13531-bib-0031] Kivinen, K. , Kallajoki, M. , & Taimen, P. (2005). Caspase‐3 is required in the apoptotic disintegration of the nuclear matrix. Experimental Cell Research, 311(1), 62–73. 10.1016/j.yexcr.2005.08.006 16199031

[acel13531-bib-0032] Klein, H.‐U. , McCabe, C. , Gjoneska, E. , Sullivan, S. E. , Kaskow, B. J. , Tang, A. , Smith, R. V. , Xu, J. , Pfenning, A. R. , Bernstein, B. E. , Meissner, A. , Schneider, J. A. , Mostafavi, S. , Tsai, L.‐H. , Young‐Pearse, T. L. , Bennett, D. A. , & De Jager, P. L. (2019). Epigenome‐wide study uncovers large‐scale changes in histone acetylation driven by tau pathology in aging and Alzheimer's human brains. Nature Neuroscience, 22(1), 37–46. 10.1038/s41593-018-0291-1 30559478 PMC6516529

[acel13531-bib-0033] Lieberman‐Aiden, E. , van Berkum, N. L. , Williams, L. , Imakaev, M. , Ragoczy, T. , Telling, A. , Amit, I. , Lajoie, B. R. , Sabo, P. J. , Dorschner, M. O. , Sandstrom, R. , Bernstein, B. , Bender, M. A. , Groudine, M. , Gnirke, A. , Stamatoyannopoulos, J. , Mirny, L. A. , Lander, E. S. , & Dekker, J. (2009). Comprehensive mapping of long‐range interactions reveals folding principles of the human genome. Science, 326(5950), 289–293. 10.1126/science.1181369 19815776 PMC2858594

[acel13531-bib-0034] Lindenboim, L. , Zohar, H. , Worman, H. J. , & Stein, R. (2020). The nuclear envelope: Target and mediator of the apoptotic process. Cell Death Discovery, 6, 29. 10.1038/s41420-020-0256-5 32351716 PMC7184752

[acel13531-bib-0035] Long, J. M. , & Holtzman, D. M. (2019). Alzheimer disease: An update on pathobiology and treatment strategies. Cell, 179(2), 312–339. 10.1016/j.cell.2019.09.001 31564456 PMC6778042

[acel13531-bib-0036] Lovell, M. A. , Xie, C. , Gabbita, S. P. , & Markesbery, W. R. (2000). Decreased thioredoxin and increased thioredoxin reductase levels in Alzheimer's disease brain. Free Radical Biology and Medicine, 28(3), 418–427. 10.1016/s0891-5849(99)00258-0 10699754

[acel13531-bib-0037] Luo, Q. , Lin, Y. X. , Yang, P. P. , Wang, Y. , Qi, G. B. , Qiao, Z. Y. , & Wang, H. (2018). A self‐destructive nanosweeper that captures and clears amyloid beta‐peptides. Nature Communications, 9(1), 1802. 10.1038/s41467-018-04255-z PMC5935695 29728565

[acel13531-bib-0038] Malhas, A. N. , Lee, C. F. , & Vaux, D. J. (2009). Lamin B1 controls oxidative stress responses via Oct‐1. Journal of Cell Biology, 184(1), 45–55. 10.1083/jcb.200804155 PMC2615091 19139261

[acel13531-bib-0039] Mi, W. , Pawlik, M. , Sastre, M. , Jung, S. S. , Radvinsky, D. S. , Klein, A. M. , & Levy, E. (2007). Cystatin C inhibits amyloid‐beta deposition in Alzheimer's disease mouse models. Nature Genetics, 39(12), 1440–1442. 10.1038/ng.2007.29 18026100

[acel13531-bib-0040] Montaser, M. , Lalmanach, G. , & Mach, L. (2002). CA‐074, but not its methyl ester CA‐074Me, is a selective inhibitor of cathepsin B within living cells. Biological Chemistry, 383(7–8), 1305–1308. 10.1515/BC.2002.147 12437121

[acel13531-bib-0041] Mueller‐Steiner, S. , Zhou, Y. , Arai, H. , Roberson, E. D. , Sun, B. , Chen, J. , Wang, X. , Yu, G. , Esposito, L. , Mucke, L. , & Gan, L. I. (2006). Antiamyloidogenic and neuroprotective functions of cathepsin B: Implications for Alzheimer's disease. Neuron, 51(6), 703–714. 10.1016/j.neuron.2006.07.027 16982417

[acel13531-bib-0042] Nixon, R. A. (2013). The role of autophagy in neurodegenerative disease. Nature Medicine, 19(8), 983–997. 10.1038/nm.3232 23921753

[acel13531-bib-0043] Nixon, R. A. (2017). Amyloid precursor protein and endosomal‐lysosomal dysfunction in Alzheimer's disease: Inseparable partners in a multifactorial disease. The FASEB Journal, 31(7), 2729–2743. 10.1096/fj.201700359 28663518 PMC6137496

[acel13531-bib-0044] Noonan, J. , Tanveer, R. , Klompas, A. , Gowran, A. , McKiernan, J. , & Campbell, V. A. (2010). Endocannabinoids prevent beta‐amyloid‐mediated lysosomal destabilization in cultured neurons. Journal of Biological Chemistry, 285(49), 38543–38554. 10.1074/jbc.M110.162040 PMC2992287 20923768

[acel13531-bib-0045] Oddo, S. , Caccamo, A. , Shepherd, J. D. , Murphy, M. P. , Golde, T. E. , Kayed, R. , Metherate, R. , Mattson, M. P. , Akbari, Y. , & LaFerla, F. M. (2003). Triple‐transgenic model of Alzheimer's disease with plaques and tangles: Intracellular Abeta and synaptic dysfunction. Neuron, 39(3), 409–421. 10.1016/s0896-6273(03)00434-3 12895417

[acel13531-bib-0046] Perlenfein, T. J. , & Murphy, R. M. (2017). A mechanistic model to predict effects of cathepsin B and cystatin C on beta‐amyloid aggregation and degradation. Journal of Biological Chemistry, 292(51), 21071–21082. 10.1074/jbc.M117.811448 PMC5743080 29046353

[acel13531-bib-0047] Raffel, J. , Bhattacharyya, A. K. , Gallegos, A. , Cui, H. , Einspahr, J. G. , Alberts, D. S. , & Powis, G. (2003). Increased expression of thioredoxin‐1 in human colorectal cancer is associated with decreased patient survival. Journal of Laboratory and Clinical Medicine, 142(1), 46–51. 10.1016/S0022-2143(03)00068-4 12878985

[acel13531-bib-0048] Ramasamy, V. S. , Islam, M. I. , Haque, M. A. , Shin, S. Y. , & Park, I. S. (2016). beta‐Amyloid induces nuclear protease‐mediated lamin fragmentation independent of caspase activation. Biochimica et Biophysica Acta, 1863(6 Pt A), 1189–1199. 10.1016/j.bbamcr.2016.02.008 26876308

[acel13531-bib-0049] Rao, L. , Perez, D. , & White, E. (1996). Lamin proteolysis facilitates nuclear events during apoptosis. Journal of Cell Biology, 135(6 Pt 1), 1441–1455. 10.1083/jcb.135.6.1441 PMC2133948 8978814

[acel13531-bib-0050] Reddy, K. L. , Zullo, J. M. , Bertolino, E. , & Singh, H. (2008). Transcriptional repression mediated by repositioning of genes to the nuclear lamina. Nature, 452(7184), 243–247. 10.1038/nature06727 18272965

[acel13531-bib-0051] Righolt, C. H. , Guffei, A. , Knecht, H. , Young, I. T. , Stallinga, S. , van Vliet, L. J. , & Mai, S. (2014). Differences in nuclear DNA organization between lymphocytes, Hodgkin and Reed‐Sternberg cells revealed by structured illumination microscopy. Journal of Cellular Biochemistry, 115(8), 1441–1448. 10.1002/jcb.24800 24590512 PMC4231252

[acel13531-bib-0052] Schirmer, E. C. , & Gerace, L. (2004). The stability of the nuclear lamina polymer changes with the composition of lamin subtypes according to their individual binding strengths. Journal of Biological Chemistry, 279(41), 42811–42817. 10.1074/jbc.M407705200 15284226

[acel13531-bib-0053] Selznick, L. A. , Zheng, T. S. , Flavell, R. A. , Rakic, P. , & Roth, K. A. (2000). Amyloid beta‐induced neuronal death is bax‐dependent but caspase‐independent. Journal of Neuropathology and Experimental Neurology, 59(4), 271–279. 10.1093/jnen/59.4.271 10759182

[acel13531-bib-0054] Stover, K. R. , Campbell, M. A. , Van Winssen, C. M. , & Brown, R. E. (2015). Early detection of cognitive deficits in the 3xTg‐AD mouse model of Alzheimer's disease. Behavioral Brain Research, 289, 29–38. 10.1016/j.bbr.2015.04.012 25896362

[acel13531-bib-0055] Sun, W. , Samimi, H. , Gamez, M. , Zare, H. , & Frost, B. (2018). Pathogenic tau‐induced piRNA depletion promotes neuronal death through transposable element dysregulation in neurodegenerative tauopathies. Nature Neuroscience, 21(8), 1038–1048. 10.1038/s41593-018-0194-1 30038280 PMC6095477

[acel13531-bib-0056] Tamhane, T. , Lllukkumbura, R. , Lu, S. , Maelandsmo, G. M. , Haugen, M. H. , & Brix, K. (2016). Nuclear cathepsin L activity is required for cell cycle progression of colorectal carcinoma cells. Biochimie, 122, 208–218. 10.1016/j.biochi.2015.09.003 26343556

[acel13531-bib-0057] Vaz, M. , & Silvestre, S. (2020). Alzheimer's disease: Recent treatment strategies. European Journal of Pharmacology, 887, 173554. 10.1016/j.ejphar.2020.173554 32941929

[acel13531-bib-0058] Venojärvi, M. , Korkmaz, A. , Aunola, S. , Hällsten, K. , Virtanen, K. , Marniemi, J. , Halonen, J.‐P. , Hänninen, O. , Nuutila, P. , & Atalay, M. (2014). Decreased thioredoxin‐1 and increased HSP90 expression in skeletal muscle in subjects with type 2 diabetes or impaired glucose tolerance. BioMed Research International, 2014, 386351. 10.1155/2014/386351 24689038 PMC3932292

[acel13531-bib-0059] Wang, C. , Sun, B. , Zhou, Y. , Grubb, A. , & Gan, L. (2012). Cathepsin B degrades amyloid‐beta in mice expressing wild‐type human amyloid precursor protein. Journal of Biological Chemistry, 287(47), 39834–39841. 10.1074/jbc.M112.371641 PMC3501032 23024364

[acel13531-bib-0060] Wang, Y. , Wang, Y. , Bharti, V. , Zhou, H. , Hoi, V. , Tan, H. , & Wang, J. F. (2019). Upregulation of thioredoxin‐interacting protein in brain of amyloid‐beta protein precursor/presenilin 1 transgenic mice and amyloid‐beta treated neuronal cells. Journal of Alzheimer's Disease, 72(1), 139–150. 10.3233/JAD-190223 31561358

[acel13531-bib-0061] Young, S. G. , Jung, H. J. , Lee, J. M. , & Fong, L. G. (2014). Nuclear lamins and neurobiology. Molecular and Cellular Biology, 34(15), 2776–2785. 10.1128/MCB.00486-14 24842906 PMC4135577

[acel13531-bib-0062] Zhang, D. , Beresford, P. J. , Greenberg, A. H. , & Lieberman, J. (2001). Granzymes A and B directly cleave lamins and disrupt the nuclear lamina during granule‐mediated cytolysis. Proceedings of the National Academy of Sciences of the United States of America, 98(10), 5746–5751. 10.1073/pnas.101329598 11331782 PMC33284

[acel13531-bib-0063] Zheng, X. , Chu, F. , Mirkin, B. L. , Sudha, T. , Mousa, S. A. , & Rebbaa, A. (2008). Role of the proteolytic hierarchy between cathepsin L, cathepsin D and caspase‐3 in regulation of cellular susceptibility to apoptosis and autophagy. Biochimica et Biophysica Acta, 1783(12), 2294–2300. 10.1016/j.bbamcr.2008.07.027 18775751

[acel13531-bib-0064] Zhou, F. , Xiong, X. , Li, S. , Liang, J. , Zhang, X. , Tian, M. , & Li, Y. (2020). Enhanced autophagic retrograde axonal transport by dynein intermediate chain upregulation improves Abeta clearance and cognitive function in APP/PS1 double transgenic mice. Aging (Albany NY), 12(12), 12142–12159. 10.18632/aging.103382 32584265 PMC7343509

[acel13531-bib-0065] Zhou, L. , Flores, J. , Noel, A. , Beauchet, O. , Sjostrom, P. J. , & LeBlanc, A. C. (2019). Methylene blue inhibits Caspase‐6 activity, and reverses Caspase‐6‐induced cognitive impairment and neuroinflammation in aged mice. Neuropathologica Communications, 7(1), 210. 10.1186/s40478-019-0856-6 PMC6915996 31843022

[acel13531-bib-0066] Zullo, J. M. , Demarco, I. A. , Piqué‐Regi, R. , Gaffney, D. J. , Epstein, C. B. , Spooner, C. J. , Luperchio, T. R. , Bernstein, B. E. , Pritchard, J. K. , Reddy, K. L. , & Singh, H. (2012). DNA sequence‐dependent compartmentalization and silencing of chromatin at the nuclear lamina. Cell, 149(7), 1474–1487. 10.1016/j.cell.2012.04.035 22726435

