# Supplementary figures and images for: Regulatory role of cathepsin L in induction of nuclear laminopathy in Alzheimer’s disease
Source: Aging Cell. 2021 Dec 14;21(1):e13531. doi: 10.1111/acel.13531 (PMC8761039; doi:10.1111/acel.13531)

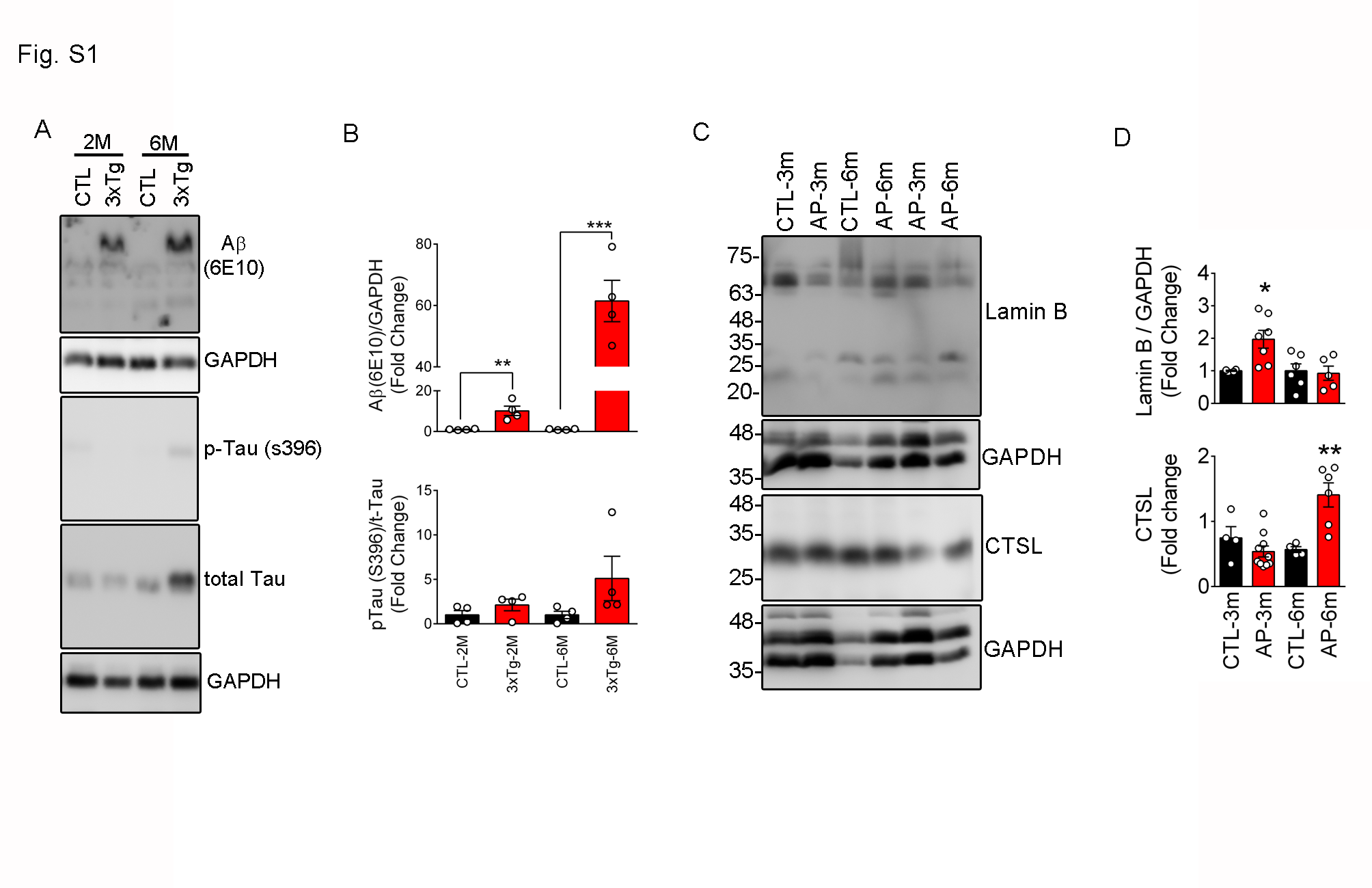

Supplement: Supplementary file 1 — Figure S1 [file ACEL-21-e13531-s004.tif]

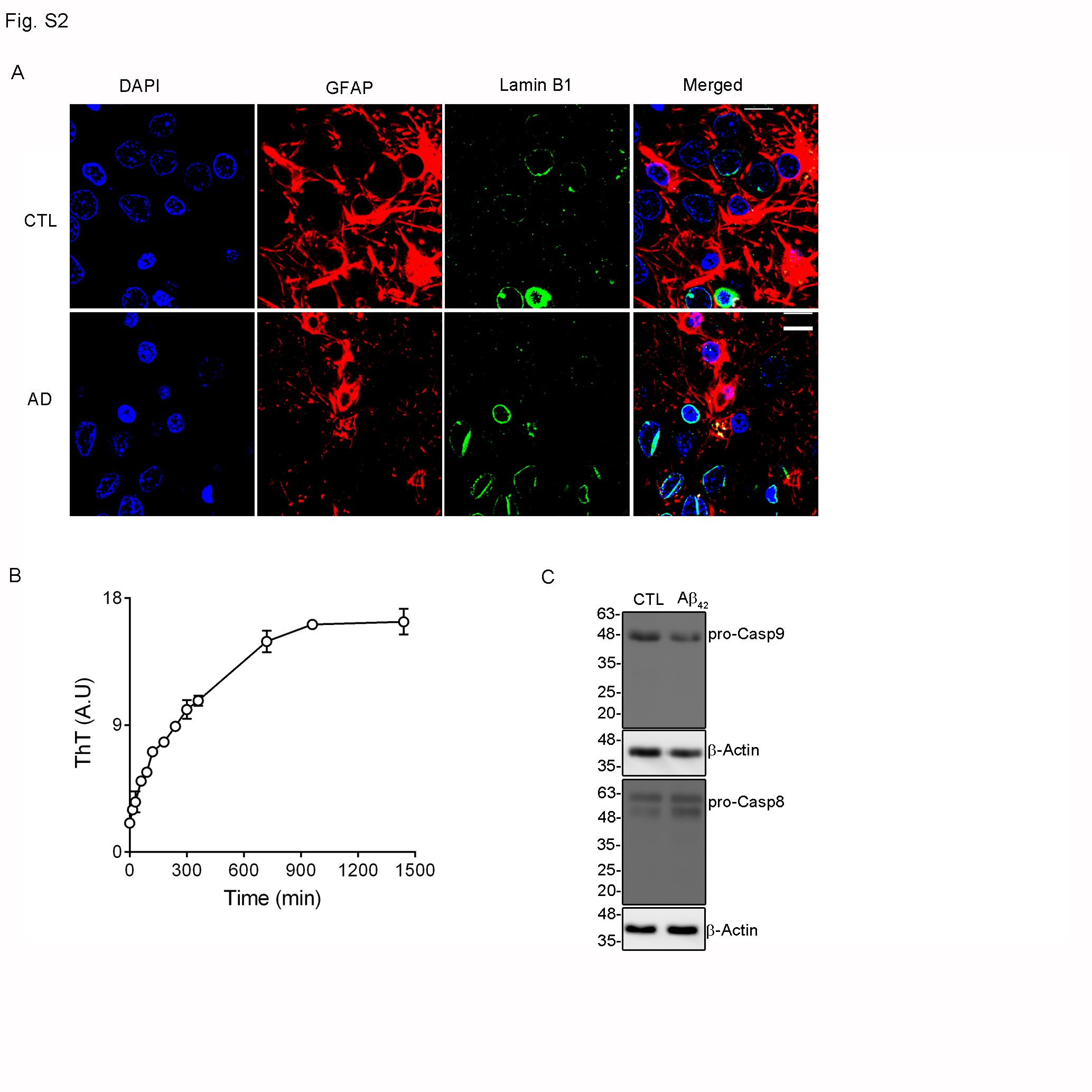

Supplement: Supplementary file 2 — Figure S2 [file ACEL-21-e13531-s006.tif]

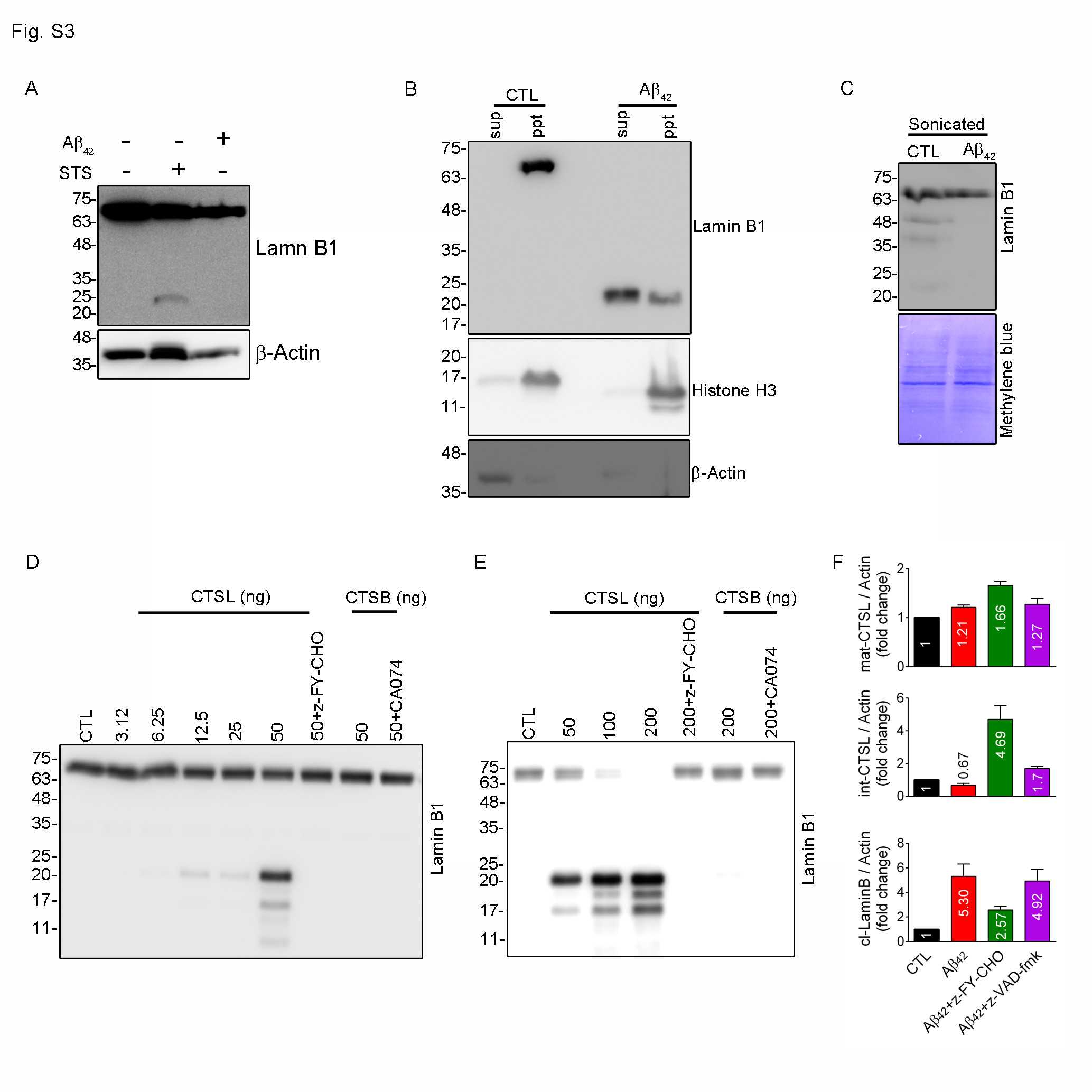

Supplement: Supplementary file 3 — Figure S3 [file ACEL-21-e13531-s005.tif]

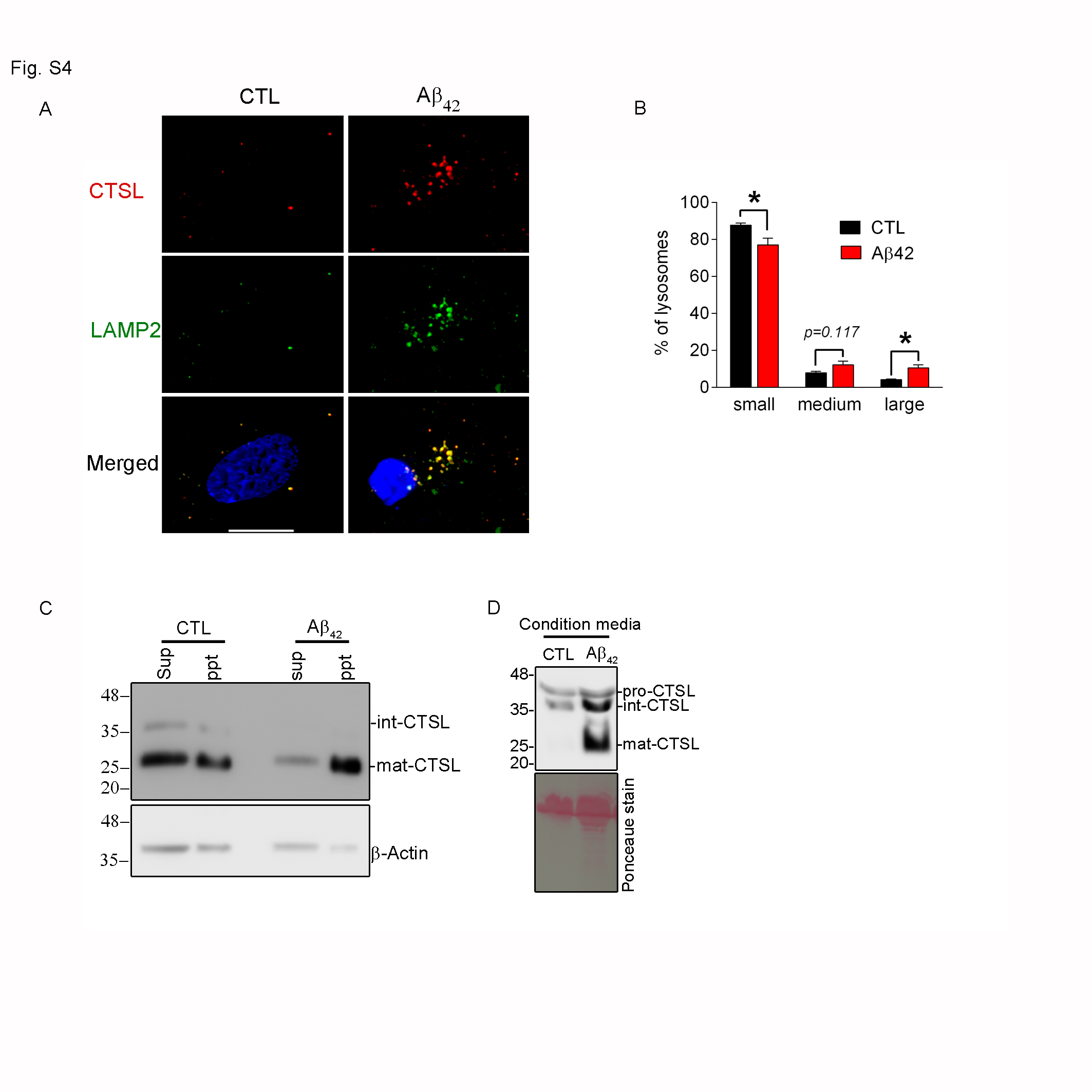

Supplement: Supplementary file 4 — Figure S4 [file ACEL-21-e13531-s001.tif]

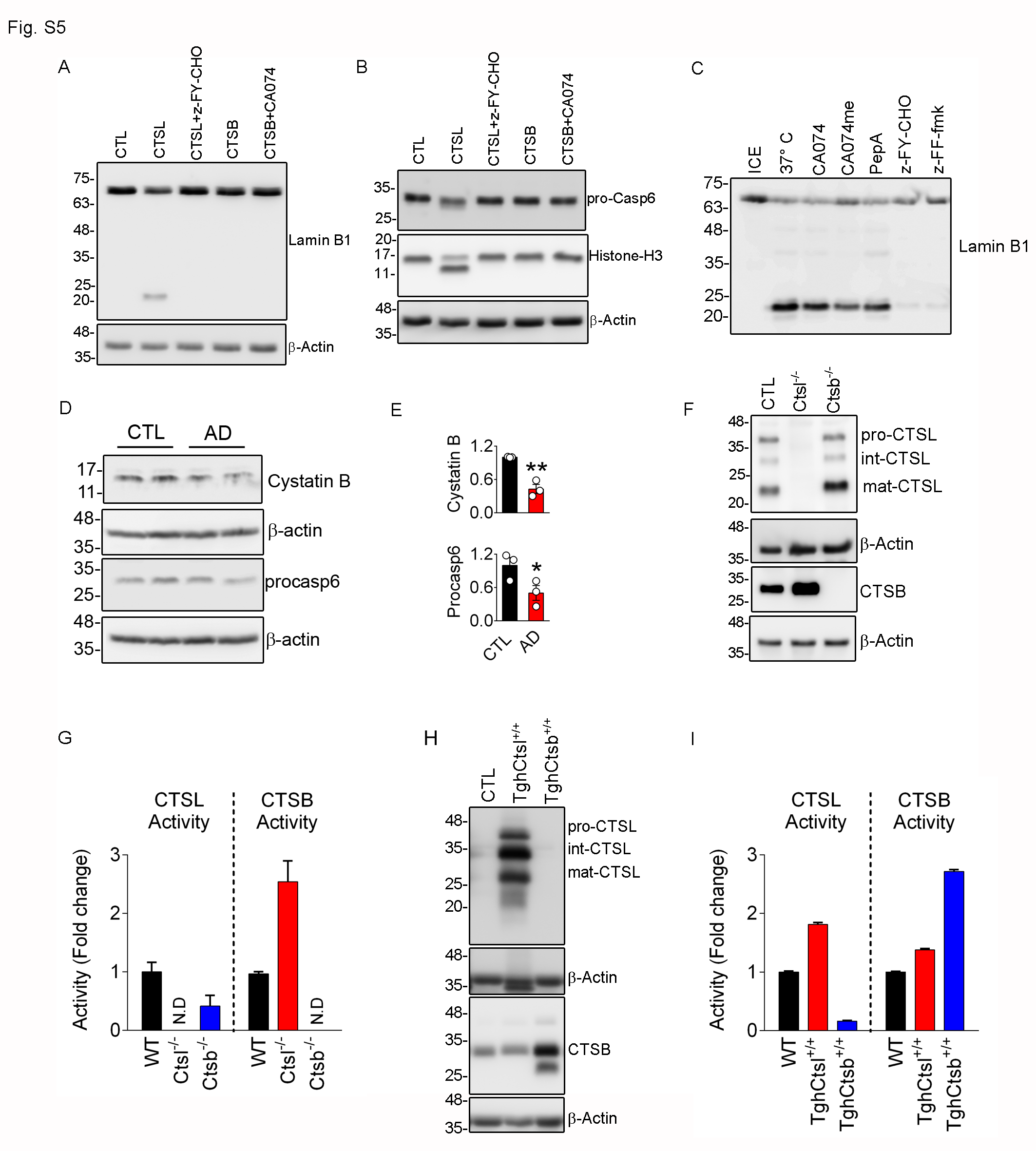

Supplement: Supplementary file 5 — Figure S5 [file ACEL-21-e13531-s003.tif]

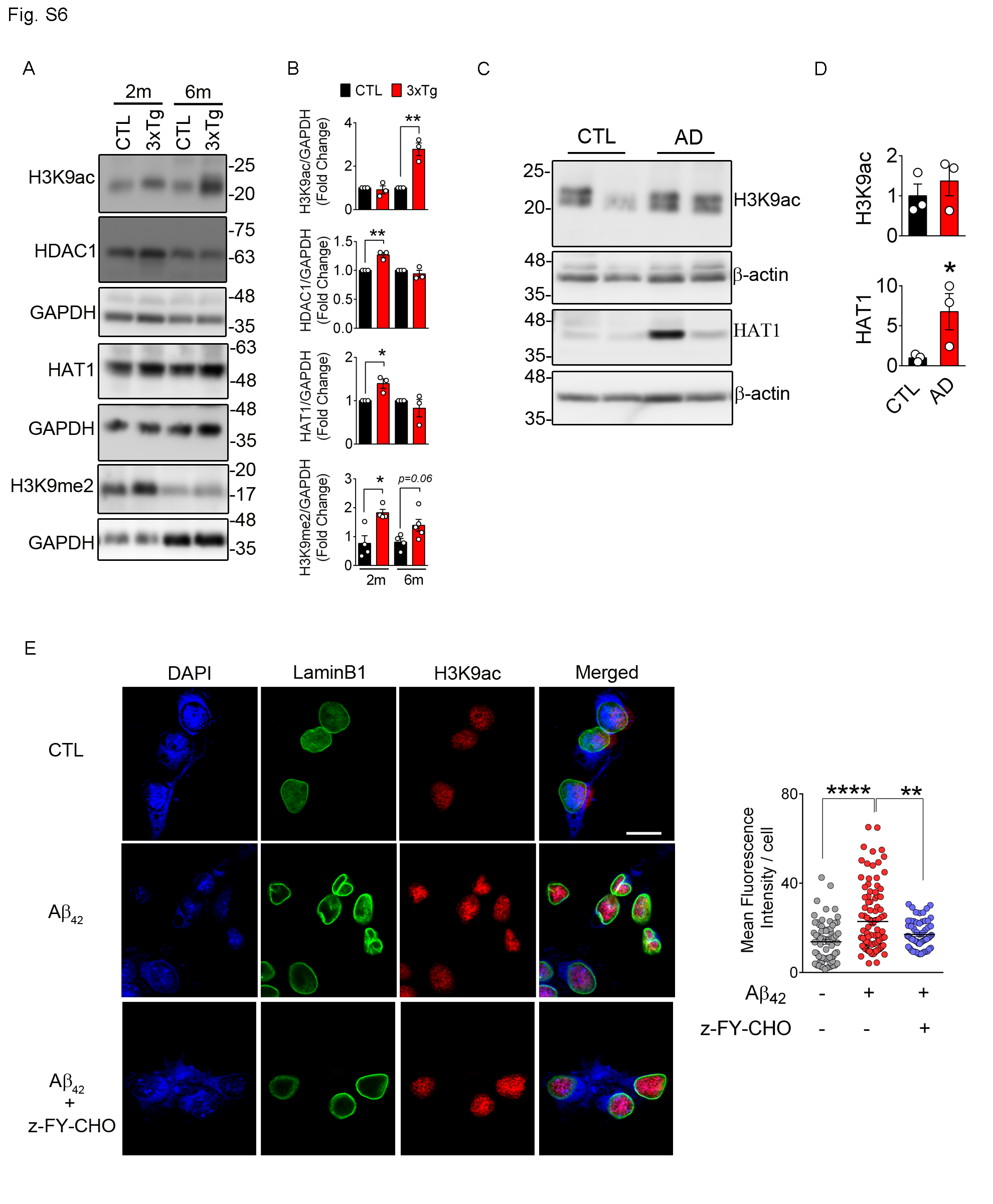

Supplement: Supplementary file 6 — Figure S6 [file ACEL-21-e13531-s007.tif]
