# Supplementary material for: Regulatory role of cathepsin L in induction of nuclear laminopathy in Alzheimer’s disease
Source: Aging Cell. 2021 Dec 14;21(1):e13531. doi: 10.1111/acel.13531 (PMC8761039; doi:10.1111/acel.13531)
Supplement: Supplementary file 7 — Tables S1–S3 [file ACEL-21-e13531-s002.docx]

**Regulatory role of cathepsin L in induction of nuclear laminopathy in Alzheimer’s Disease**

Md Imamul Islam^1,9^, Pandian Nagakannan^1,9^, Tetiana Shcholok^1,9^, Fabio Contu^4^, Sabine Mai^4^, Benedict C Albensi^3,8,9^, Marc R Del Bigio^5,9^, Jun-Feng, Wang^6,9^, Md Golam Sharoar^7^, Riqiang Yan^7^, Il-Seon Park^2^, Eftekhar Eftekharpour^1,9^*

^1^Department of Physiology and Pathophysiology, University of Manitoba, Winnipeg, Canada, ^2^Department of Cellular and Molecular Medicine, Chosun University, Gwangju, South Korea, ^3^St Boniface Hospital, Albrechtsen Research Centre, Winnipeg, Canada, ^4^Cell Biology, Research Institute of Oncology and Hematology, University of Manitoba, CancerCare Manitoba, Winnipeg, Canada, ^5^Dept. of Pathology and Shared Health Manitoba, , ^6^Department of Pharmacology and Therapeutics, University of Manitoba, Canada, ^7^Department of Neuroscience, University of Connecticut Health, Farmington, Connecticut 06032, USA, ^8^Deprtment of Pharmaceutical Sciences, College of Pharmacy, Nova Southern University, ^9^Rady Faculty of Health Sciences, University of Manitoba, Winnipeg, Canada.

*** Corresponding Author:**

Eftekhar Eftekharpour

Department of Physiology and Pathophysiology, University of Manitoba, Winnipeg, Canada

# [Eftekhar.eftekharpour@umanitoba.ca](mailto:Eftekhar.eftekharpour@umanitoba.ca)

**Supplemental Experimental Methods**

**Preparation of Aβ42**

Forty-two amino-acids recombinant human Aβ42 was purified according to previously published protocol ([Shahnawaz, Thapa, & Park, 2007](#_ENREF_8)). Lyophilized Aβ42 powder was dissolved in 0.1% NH4OH at a concentration of 440 µM and sonicated using an ultra-sonicator for 15 s (5s each, 3 times with an interval of 10s). Freshly prepared Aβ42 was then diluted in serum free DMEM/F-12 (1:1) media for SH-SY5Y cells and in Neurobasal A media for primary hippocampal neurons as per the desired concentration.

**Immunohistochemistry with human hippocampal tissue samples**

Research on human organ/brain tissues has been reviewed and approved by the University of Manitoba Bannatyne Campus research ethics board and Health Research Board protocol. Paraffin embedded human hippocampal tissue sections were prepared from autopsy specimens. Alzheimer disease was diagnosed using the National Institute on Aging–Alzheimer’s Association guidelines ([Montine et al., 2012](#_ENREF_4)). Anonymized tissue samples acquired from autopsies that included unrestricted consent for research were used. Intervals between death and autopsy ranged between 7 and 24 hours. Brain samples were frozen or fixed in 10% buffered formalin for 14-20 days followed by paraffin embedding. Details of the samples is available in supplementary table S3a. Immunohistochemistry was performed as reported previously ([Moreno-Jimenez et al., 2019](#_ENREF_5)). Briefly, paraffin embedded hippocampal sections (on slides) were treated with 100 % xylene for 3 times 10 min each. Then slides were treated with series of ethanol gradient 100%, 95% and 70% for two times 5 min each and finally rinsed with double distilled water. Slides were then incubated with 0.5% sodium borohydride (NaBH_4_, Sigma-Aldrich, 213462) in 0.01M sodium citrate buffer (pH6.0) for 30 min at RT followed by 5 rinses with 1x PBS. Next, samples were exposed to short intervals of 15-20s (5 times) microwave heating in 0.01M sodium citrate buffer (pH 6.0). To this end, samples were tightly closed and boiled in a water bath for 45 min in 0.01M citrate buffer only. After cooling the samples at RT for 1h, they were washed 5 times with 1x PBS. Permeabilization of the samples were done with 0.5% Triton X-100 in 1x PBS for 15 min. Afterward, blocking was performed using 1% BSA with 0.3% Triton X-100. Samples were incubated with primary antibody in 1% BSA with 0.3% Triton X-100 for 5 days at 4° C with gentle shaking. Secondary antibody with a proper fluorescent tag was used for 24 h at 4° C. To avoid auto fluorescence, tissue sections were treated with the Vector TrueVIEW auto fluorescence quenching kit SP-8400 (Vector Laboratories, CA, USA). Images were acquired using confocal microscopy LSM 710 (Zeiss, Germany).

**Western blot with human hippocampal tissue samples**

Autopsy brain hippocampal samples were obtained from the following sources: Department of Pathology, University of Manitoba, Emory University and Cleveland Clinic Foundation tissue bank. Sample details are enlisted in supplementary table S3b. Tissues were weighed and sliced into small pieces. Lysis buffer containing 50 mM Tris-HCl pH 8.0, 150 mM NaCl, 5 mM EDTA, 5mM EGTA, 1% v/v NP-40, 1% v/v TritonX-100 with protease phosphatase inhibitor was added to the samples and homogenized with glass mortar and Teflon pestle keeping the mortar in ice. After incubating on ice for 30 min, the homogenates were centrifuged for 30 min at 15000 X g at 4° C. Supernatant was further centrifuged for 30 min at 15000 X g at 4° C. The supernatant was used for western blot analysis. Only for two control samples tissues were freshly dissected, all other samples were dissected from frozen half brains).

**Cell Culture**

SH-SY5Y human neuroblastoma cells were cultured in DMEM/F-12 medium supplemented with 10% FBS, and 1% penicillin, streptomycin and neomycin at 37 ⁰C and 5% CO2 in a humidified incubator. Primary mouse embryonic fibroblasts (MEF) wild type (WT), cathepsin B (Ctsb^-/-^) and L (Ctsl/^-/-^) knocked out, cathepsin B (Ctsb^+/+^) and L (Ctsl^+/+^) overexpressing cells were generously provided by Dr. Thomas Reinheckel, University of Freiburg, Germany ([Sevenich et al., 2011](#_ENREF_7); [Tholen et al., 2014](#_ENREF_10); [Tholen et al., 2015](#_ENREF_11)). Wild type and overexpressing human cathepsin B and human cathepsin L MEF cells were immortalized by transfecting pCMV-G-Ag plasmid which expresses T-antigen, at the Central Animal Core Imaging and Transgenic Facilities, Health Sciences Centre, University of Manitoba. To maintain the same culture conditions, MEF cells were also cultured in DMEM/F-12 media as mentioned for SH-SY5Y cells. When cells were ~70% confluent, inhibitors were diluted in DMEM/F-12 media without FBS and treated overnight. Then the culture media was completely changed with freshly prepared Aβ42/vehicle at the indicated concentration and duration.

Primary hippocampal neurons were obtained from day E18 embryos of Sprague-Dawley (SD) rats. The hippocampi were dissected out and kept in artificial cerebrospinal fluid (aCSF) on ice. After washing twice with aCSF, the tissue was cut into small pieces and resuspended in aCSF (1 ml of aCSF/3 embryos) containing papain (1:1000) and incubated at 37°C for 15 min with intermittent gentle shaking. An equal volume of FBS was added to aCSF to neutralize the protease activity and centrifuged at 900 rpm for 5 min and the supernatant was discarded. Pellets were resuspended in aCSF with 1X DNAase (Sigma# D5025) and triturated with a polished Pasteur pipette for 20 times and centrifuged at 900 rpm for 5 min. The pelleted cells were resuspended in Neurobasal A (Gibco# 10,888–022) media containing 1% B27 supplement (Gibco# 17504-044), PSN and L-glutamine. Cells were counted and seeded in a PDL coated 6 well plate at a density of 150,000 cells/well. Half of the growth medium was changed every 3 days. After 10 days, cells were treated with 5 µM of Aβ42 for 16 h. For immunocytochemistry, cells were seeded on PDL coated glass cover slips. Mouse cortical neurons were collected and cultured from E-18 embryos from CD-1 mice following the same protocol used for the rat hippocampal neurons.

**Cell lysis and Western blot analysis**

After harvesting, cells were washed twice with PBS and resuspended in buffer containing 50 mM Tris-HCl pH 8.0, 150 mM NaCl, 5 mM EDTA, 1% v/v NP-40 and protease phosphatase inhibitors and incubated on ice for 30 min. Cells were then subjected to ultra-sonication for 3 cycles, 5s each with 20s interval. Supernatant obtained after micro centrifugation for 15 min at 4 ⁰C at 13000 rpm, was collected and measured for protein concentration using a BCA protein assay kit (Bio-Rad Laboratories Ltd, Missisauga, ON). Samples for immunoblots were prepared and loaded in 4-13% SDS-PAGE and transferred to a PVDF membrane. Membranes were blocked with blocking buffer (5% non-fat milk in Tris buffered saline with 0.2% of Tween 20) and incubated with appropriate primary antibodies overnight at 4 ⁰C. On the next day, membranes were washed and incubated with HRP-conjugated secondary antibodies at room temperature for 1 h. Proteins were detected using ECL (Bio-Rad Laboratories Ltd, Missisauga, ON). Actin and GAPDH were used as a loading control. Densitometric analysis were carried out using AlphaEaseFC (version 6.0.0 Alpha Innotech).

**Immunocytochemistry**

Cells were cultured and treated on German glass cover slips thickness #1.5 for super resolution 3D structured illuminated microscopy and 3D confocal microscopy. Cells were then fixed with 3.7% of paraformaldehyde in 1x PBS for 10 min at room temperature (RT) followed by washing 3 times 5 min each in 1x PBS. After permeabilizing with 0.3% Triton-X 100 in blocking buffer (1% BSA in 1x PBS) at RT for 1 h, coverslips were incubated with appropriate primary antibodies overnight at 4° C. Next day, samples were washed and incubated with fluorescent tagged secondary antibodies followed by counterstaining with 4′,6-diamidino-2-phenylindole (DAPI). Samples were mounted with Vectasheild mounting media. Coverslips were imaged using Zeiss AxioImager Z1 microscope (Carl Zeiss, Toronto, ON, Canada) fitted with an AzioCam HRm camera with a 63x/1.4 oil plan apochromat objective lens using the imaging software Axiovision 4.8.2 (Zeiss). 3D image acquisition was obtained using 40 z stacks of 0.2 µm (z plane) with a distance of 102 nm in both x- and y- plane. The exposure time of Cy3 and FITC was maintained constant whereas exposure time for DAPI was adjusted with different images. Finally, images were deconvoluted using Axiovision 4.8.2 software. More than 30 individual cells were analyzed for each cover slips.

**Assessment of Enzymatic Activity**

Cathepsin B and L activities were measured by using fluorometric synthetic substrates ([Lee et al., 2017](#_ENREF_3)) without any pre-incubation. Tissue or cell lysates (without protease inhibitors) were diluted in reaction buffer (25 mM MES pH 5.0, 5 mM DTT, 1 mM EDTA) in a 96 black well plate. In cathepsin L activity assay, 10 µL of 50 µM CA074 was used to inhibit cathepsin B activity so that activity shown by substrate was specific to cathepsin L. Fifty µL of 200 µM Z-Phe-Arg-AMC diluted in reaction buffer was then added to the reaction mixture final volume of 100 µL to initiate the reaction. For cathepsin B activity, 50 µL of 100 µM Z-Arg-Arg-AMC was added to the reaction mixture. Release of AMC as function of cathepsin activity was measured kinetically at excitation and emission wavelengths of 360/480 for 45 min at 1 min interval in a BioTek H1-Synergy microplate reader (Winnoski, VT, United States). Slopes were expressed as arbitrary units. The effect of pH 7.4 on cathepsin L activity was assessed using the following reaction buffer: 20 mM HEPES-KOH pH 7.4, 5 mM DTT, 1 mM EDTA. Caspase-3 and -6 activity was measured by using Ac-DEVD-amc and Ac-VEID-amc as substrate respectively in caspase assay buffer (20 mM HEPES-NaOH, pH 7.0, 20 mM NaCl, 1.5 mM MgCl2, 1 mM EDTA, 1 mM EGTA and 10 mM DTT).

**Acridine Orange Lysosomal staining**

After treatment with Aβ42, cells were washed with serum free DMEM/F-12 media once and were incubated with acridine orange (2 µg/ml) containing media at 37 °C for 15 min. The reaction was terminated by washing the cells twice with serum free and phenol red free DMEM/F-12 media, followed by immediate imaging using a LSM710 Zeiss confocal microscope (Zeiss, Germany). Quantification of green fluorescence emitted by acridine orange was measured using image J software (NIH, version 1.49v).

**Sub-Cellular Fractionation**

A mild lysis buffer (digitonin buffer, containing 75 mM NaCl, 1 mM NaH2PO4, 8 mM Na2HPO4, 250 mM sucrose and 190 μg/ml digitonin) was used for cell fractionation after Aβ42 treatment (5 µM, 16 h) ([Sharoar, Islam, Shahnawaz, Shin, & Park, 2014](#_ENREF_9)). Cells were kept on ice for 5 min in digitonin buffer, then centrifuged at 13000 rpm (5 min at 4 °C). The soluble supernatant (sup) was collected and the precipitant (ppt) was washed with digitonin buffer and was re-dissolved in NP-40 containing buffer (50 mM Tris-HCl (pH 8.0), 150 mM NaCl, 5 mM EDTA, 1% v/v NP-40 buffer). Samples were mixed by pipetting and kept on ice for 30 min followed by centrifugation at 13000 rpm for 30 min at 4 °C. The supernatant was collected, and the precipitant was again dissolved in the above-mentioned lysis buffer (NP-40) and then sonicated for 15 seconds (3x5s, 30% amplification). The resulting supernatant (termed as sonicated) was collected after centrifuging at 13000 rpm for 30 min at 4 °C.

**Three-Dimensional Structured Illuminated Microscopy (3D SIM) and Granulometry**

A Zeiss ELYRA PS1 (Zeiss, Toronto, Ontario, Canada) equipped with a PlanApochromat 63×/1.40 Oil immersion objective using an EMCCD iXon 885 camera and a 1.6× tube lens was employed to develop 3D-SIM images. 405 nm laser excitation with 23 μm diffraction grating and filter cube (SR Cube 07) was used for DAPI channel. The lateral pixel size, Δx and Δy, was 79 nm in the recorded images and 40 nm in the reconstructed image, the step between z-planes, Δz, was 91 nm. 3D-SIM images were reconstructed using ZEN 2012 black edition (Carl Zeiss, Jena, Germany) with the standard settings. Granulometry analysis of the images was done in Matlab with the toolbox DIPimage. The parameters were set according to a previous report ([Garcia et al., 2017](#_ENREF_1)). The DNA structure and DNA free space were measured using a morphological sieve applied to the unclipped images. DNA compaction is measured by granulometry of the positive image (light granulometry) and the inverted image/negative image is used for dark granulometry to assess the DNA-free space.

**Expression and Purification of Recombinant Proteins**

Human LB1 bacterial plasmid was a kind gift from Dr. Larry Gerace, Department of Cell Biology, The Scripps Research Institute, La Jolla, California, USA. Protein purification was done as reported earlier with minor modifications ([Schirmer & Gerace, 2004](#_ENREF_6)). BL21–DE3 bacterial cells containing human LB1 were grown at 37° C at 220 rpm until the OD_600_ value reached 0.6-0.8, then, proteins were induced using 400 µM of IPTG (isopropyl-1-thio--D-galactopyranoside) for 4 h at 37° C. Cells were collected by centrifugation and washed with STE buffer 10 mM Tris-HCl (pH8.0), 10 mM NaCl, 1 mM EDTA. Bacterial cells were lysed using ultra sonication with lysis buffer consisting of 25 mM HEPES (pH 8.0), 0.1 mM MgCl_2_, 3 mM β-mercaptoethanol, 1 mM PMSF. The lysate was spun, and pellets were separated from supernatant and washed with lysis buffer twice. Pellets were solubilized in solubilization buffer 20 mM HEPES (pH 8.0), 6 M Urea, 2 mM β-mercaptoethanol. Protein samples were then injected to nickel affinity column and washed with four column volume of solubilisation buffer containing 10 mM and 20 mM imidazole twice. Finally, protein was eluted with 250 mM of imidazole in solubilisation buffer. A PD-10 column (GE17-0851-01: GE Healthcare, Buckinghamshire, UK) was employed to remove imidazole. Protein was quantified and stored at -80° C until use. CASP6 protein was purified as described previously ([Islam et al., 2019](#_ENREF_2)). The biological activity of these recombinant human proteins was tested in cell free systems as later described in the results section. LB1 was incubated with indicated concentration of purified recombinant CASP6, CTSL and CTSB enzyme without/with their specific inhibitors for the specified time period in reaction buffer containing 20 mM HEPES-NaOH, pH 7.0, 20 mM NaCl, 1.5 mM MgCl2, 1 mM EDTA, 1 mM EGTA and 5 mM DTT. At the end of the reaction 500 ng of reaction mixture (based on LB1 content) was used for western blotting.

To investigate the effect of cathepsins on CASP6 cleavage, recombinant human CASP6 was incubated with cathepsin L or B with/without their synthetic inhibitors, as indicated in the figure legends. Samples were used for western blotting at the termination of enzymatic reaction.

**Mass Spectrometry**

Purified recombinant human lamin B1 (rhLB1) (100 ng/µl) was incubated with recombinant human rhCTSL (1.25 ng/µl) in buffer containing 20 mM of HEPES (pH 7.4), 1 mM EDTA and 5 mM DTT for 30 min at 37 °C. The reaction was stopped using z-FY-CHO (20 µM). Protein digest was desalted using a SOLA HRP cartridge (ThermoFisher Scientific) and lyophilized. Dried samples were stored at -20˚C until MS analysis at the Manitoba Centre for Proteomics and Systems Biology as detailed below.

The peptide mixture was resuspended in 0.1% formic acid for LC-MS analysis with Orbitrap Q Exactive HF-X instrument (Thermo Fisher Scientific, Bremen, Germany). Samples were introduced using an Easy-nLC 1000 system (Thermo Fisher Scientific) at 3µl / injection. Mobile phase A was 0.1% (v/v) formic acid and mobile phase B was 0.1% (v/v) formic acid in 80% acetonitrile (LC-MS grade). Gradient separation of peptides was performed on a C18 (Luna C18(2), 3 μm particle size (Phenomenex, Torrance, CA)) column packed in-house in Pico-Frit (100 μm X 30 cm) capillaries (New Objective, Woburn, MA). Peptide was separated by running the following gradient: 2 – 6 % phase B over 3 minutes, 6 – 25 % over 40 minutes, 25-45% over 6 minutes, 45-90% over 1 minutes, with final elution of 90% B for 10 minutes at a flow rate of 300 nL/min.

The Orbitrap Q Exactive HF-X instrument was configured for data-dependent methods in a positive mode. Spray voltage was set to 1.85 kV, funnel RF level at 45, and heated capillary at 275 °C. Survey scans covering the mass range of 350–1500 m/z were acquired at a resolution of 60,000 (at m/z 200), with a maximum ion injection time of 50 milliseconds, and an automatic gain control (AGC) target value of 1e^6^. For MS2 scan triggering, up to 10 most abundant ions were selected for fragmentation at 28% normalized collision energy, with intensity threshold kept at 6.3e^4^. AGC target value for fragment spectra was set at 2e^5^, which were acquired at a resolution of 30,000, with a maximum ion injection time of 80 milliseconds and an isolation width set at 2.0 m/z. Dynamic exclusion of previously selected masses was enabled for 10 seconds, charge state filtering was limited to 2–6, peptide match was set to preferred, and isotope exclusion was on. The cleavage sites of CTSL in lamin B were identified by mapping MS, and the data were analyzed based on peptide spectrum matches (PSM) considering PSM 7 as the threshold.

# **Supplementary References**

Garcia, A., Huang, D., Righolt, A., Righolt, C., Kalaw, M. C., Mathur, S., . . . Mai, S. (2017). Super-resolution structure of DNA significantly differs in buccal cells of controls and Alzheimer's patients. *J Cell Physiol, 232*(9), 2387-2395. doi: 10.1002/jcp.25751

Islam, M. I., Nagakannan, P., Ogungbola, O., Djordjevic, J., Albensi, B. C., & Eftekharpour, E. (2019). Thioredoxin system as a gatekeeper in caspase-6 activation and nuclear lamina integrity: Implications for Alzheimer's disease. *Free Radic Biol Med, 134*, 567-580. doi: 10.1016/j.freeradbiomed.2019.02.010

Lee, C. W., Stankowski, J. N., Chew, J., Cook, C. N., Lam, Y. W., Almeida, S., . . . Petrucelli, L. (2017). The lysosomal protein cathepsin L is a progranulin protease. *Mol Neurodegener, 12*(1), 55. doi: 10.1186/s13024-017-0196-6

Montine, T. J., Phelps, C. H., Beach, T. G., Bigio, E. H., Cairns, N. J., Dickson, D. W., . . . Hyman, B. T. (2012). National Institute on Aging-Alzheimer's Association guidelines for the neuropathologic assessment of Alzheimer's disease: a practical approach. [Practice Guideline

Research Support, N.I.H., Extramural

Research Support, Non-U.S. Gov't]. *Acta Neuropathol, 123*(1), 1-11. doi: 10.1007/s00401-011-0910-3

Moreno-Jimenez, E. P., Flor-Garcia, M., Terreros-Roncal, J., Rabano, A., Cafini, F., Pallas-Bazarra, N., . . . Llorens-Martin, M. (2019). Adult hippocampal neurogenesis is abundant in neurologically healthy subjects and drops sharply in patients with Alzheimer's disease. [Research Support, Non-U.S. Gov't]. *Nat Med, 25*(4), 554-560. doi: 10.1038/s41591-019-0375-9

Schirmer, E. C., & Gerace, L. (2004). The stability of the nuclear lamina polymer changes with the composition of lamin subtypes according to their individual binding strengths. [Research Support, U.S. Gov't, P.H.S.]. *J Biol Chem, 279*(41), 42811-42817. doi: 10.1074/jbc.M407705200

Sevenich, L., Werner, F., Gajda, M., Schurigt, U., Sieber, C., Muller, S., . . . Reinheckel, T. (2011). Transgenic expression of human cathepsin B promotes progression and metastasis of polyoma-middle-T-induced breast cancer in mice. *Oncogene, 30*(1), 54-64. doi: 10.1038/onc.2010.387

Shahnawaz, M., Thapa, A., & Park, I. S. (2007). Stable activity of a deubiquitylating enzyme (Usp2-cc) in the presence of high concentrations of urea and its application to purify aggregation-prone peptides. [Research Support, Non-U.S. Gov't]. *Biochem Biophys Res Commun, 359*(3), 801-805. doi: 10.1016/j.bbrc.2007.05.186

Sharoar, M. G., Islam, M. I., Shahnawaz, M., Shin, S. Y., & Park, I. S. (2014). Amyloid beta binds procaspase-9 to inhibit assembly of Apaf-1 apoptosome and intrinsic apoptosis pathway. [Research Support, Non-U.S. Gov't]. *Biochim Biophys Acta, 1843*(4), 685-693. doi: 10.1016/j.bbamcr.2014.01.008

Tholen, M., Hillebrand, L. E., Tholen, S., Sedelmeier, O., Arnold, S. J., & Reinheckel, T. (2014). Out-of-frame start codons prevent translation of truncated nucleo-cytosolic cathepsin L in vivo. [Research Support, Non-U.S. Gov't]. *Nat Commun, 5*, 4931. doi: 10.1038/ncomms5931

Tholen, M., Wolanski, J., Stolze, B., Chiabudini, M., Gajda, M., Bronsert, P., . . . Reinheckel, T. (2015). Stress-resistant Translation of Cathepsin L mRNA in Breast Cancer Progression. *J Biol Chem, 290*(25), 15758-15769. doi: 10.1074/jbc.M114.624353

Table S1

List of Peptides detected on proteolytically cleaved rhlaminB1

| **Peptide sequence** | **Peptide location** | **PSMs** | | **Theo. MH+ [Da]** |
| --- | --- | --- | --- | --- |
| ATPVPPRMGSRAGGPTTPLSPTRLS | 4-28 | | 15 | 2504.34 |
| SRAGGPTTPLSPTR | 13-26 | | 8 | 1397.75 |
| SRAGGPTTPLSPTRLS | 13-28 | | 7 | 1597.87 |
| SLETENSALQLQVTEREEVRGRELTGLK | 52-79 | | 9 | 3185.68 |
| NYAKKESDLNGAQIKLR | 120-136 | | 8 | 1949.05 |
| YAKKESDLNGAQIKLR | 121-136 | | 15 | 1835.00 |
| AKKESDLNGAQIKLR | 122-136 | | 8 | 1671.94 |
| KKESDLNGAQIKLR | 123-136 | | 14 | 1600.90 |
| KSMYEEEINETRRKHETR | 209-226 | | 7 | 2336.14 |
| KSMYEEEINETRRKHETRLVE | 209-229 | | 11 | 2677.34 |
| RIQELEDLLAKEKDNSRRMLT | 320-340 | | 7 | 2558.37 |
| IEEIDVDGKFIRLK | 444-457 | | 7 | 1674.94 |
| KNQNSWGTGEDVKVILK | 516-532 | | 7 | 1917.01 |
| NQNSWGTGEDVKVILK | 517-532 | | 8 | 1787.93 |

Table S2a

List of Materials used

| **REAGENT or RESOURCE** | **SOURCE** | **IDENTIFIER** |
| --- | --- | --- |
| z-Val-Ala-Asp-fluoromethyl ketone (z-VAD-fmk) | R & D Systems | FMK001 |
| z-Val-Glu-Ile-Asp-fmk (z-VEID-fmk) | R & D Systems | FMK006 |
| CA074-methyl ester | Sigma-Aldrich | Cat# C5857 |
| CTSL substrate (Z-Phe-Arg-7-amido-4-methylcoumarin, Hydrochloride) | Calbiochem | Cat# 03-32-1501 |
| CTSB Substrate III, Fluorogenic ( Z-Arg-Arg-AMC, 2HCl) | Calbiochem | Cat# 219392 |
| DAPI (4′,6-Diamidino-2-phenylindole dihydrochloride) | Sigma-Aldrich | Cat# D9542 |
| NP-40 | Calbiochem | Cat# 492015 |
| Paraformaldehyde | Sigma-Aldrich | Cat# P6148 |
| Sodium Pyruvate | Sigma-Aldrich | Cat# P5280 |
| 30% Acrylamide/Bis Solution 29:1 | BIO-RAD | Cat# 1610156 |
| Clarity Max™ Western ECL Substrate | BIO-RAD | Cat# 1705062 |
| Clarity™ Western ECL Substrate | BIO-RAD | Cat# 1705061 |
| Dulbecco's Modified Eagle Medium (DMEM) | Gibco | Cat# 11960-051 |
| Dulbecco's Phosphate Buffered Saline (DBPS) | Ge Healthcare Bio-Science | Cat# SH30028 |
| Fetal Bovine Serum, qualified, Canada | Gibco | Cat# 12483-020 |
| GlutaMAX™ Supplement | Gibco | Cat# 35050-061 |
| Halt™ Protease and Phosphatase Inhibitor Single-Use Cocktail (100X) | Thermo Scientific | Cat# 78442 |
| Immun-Blot PVDF Membrane | BIO-RAD | Cat# 1620177 |
| LysoTracker™ Red DND-99 | Invitrogen | Cat# L7528 |
| Penicillin-Streptomycin-Neomycin (PSN) Antibiotic Mixture | Gibco | Cat# 15640-055 |
| Vivaspin™ 500 MWCO 5000 | Sartorius | Cat# VS0112 |
| Trans-Blot® Turbo™ 5x Transfer Buffer | BIO-RAD | Cat# 10026938 |
| Recombinant Human Cathepsin B Protein, CF | R & D Systems | Cat# 953-CY |
| Recombinant Human Cathepsin L Protein, CF | R & D Systems | Cat# 952-CY |
| Pierce™ BCA Protein Assay Kit | Thermo Scientific | Cat# 23225 |
| μ-Slide 8 well, ibiTreat | Ibidi GmbH, Germany | Cat# 80826 |
| German Glass cover slips #1.5 | Electron Microscopy Sciences, USA | Cat# 72290-04 |

Table S2b

List of antibodies and dilution used

| **ANTIBODIES** | **DILUTION** | **SOURCE** | **IDENTIFIER** | **RRID** |
| --- | --- | --- | --- | --- |
| Cathepsin B | 1;250 (ICC), 1:1000 (WB) | CST | Cat# 31718 | AB_2799564 |
| CASP6 | 1:1000 (WB) | CST | Cat# 9761 | AB_2290879 |
| cl-CASP3 | 1:1000 (WB) | CST | Cat# 9661 | AB_2341188 |
| Atg5 | 1:1000 (WB) | CST | Cat# 12994 | AB_2630393 |
| Atg7 | 1:1000 (WB) | CST | Cat# 8558 | AB_10831194 |
| Cathepsin D | 1:1000 (WB) | SCB | Cat# sc-377299 | AB_2827539 |
| Cathepsin L | 1:250 (IHC/ICC), 1:1500 (WB) | R & D Systems | Cat# AF1515 | AB_2087690 |
| Cathepsin L (human specific) | 1:200 (IHC/ICC), 1:1500 (WB) | R & D Systems | Cat# AF952 | AB_355737 |
| Cystatin B | 1:1000 (WB) | Invitrogen | Cat# PA5-42772 | AB_2609760 |
| Lamin B1 | 1:250 (IHC/ICC) | Abcam | Cat# ab16048 | AB_443298 |
| Lamin B1 | 1:1000 (WB) | SCB | Cat# sc-377000 | AB_2861346 |
| Histone H3 | 1:4000 (WB) | Abcam | Cat# ab1791 | AB_302613 |
| H3K9ac | 1:250 (ICC), 1:1000 ( WB) | Abcam | Cat# ab12179 | AB_298910 |
| H3K9me2 | 1:200 (ICC), 1:1000 (WB) | Abcam | Cat# ab32521 | AB_732927 |
| HAT1 | 1:1000 (WB) | Invitrogen | Cat# PA5-57817 | AB_2642314 |
| HDAC1 | 1:1000 (WB) | Invitrogen | Cat# PA1-860 | AB_2118091 |
| G9a | 1:1000 (WB) | CST | Cat# 3306 | AB_2097647 |
| GAPDH-HRP | 1:5000 (WB) | SCB | Cat# sc-166574 | AB_2107296 |
| LAMP-2 | 1:1000 (WB), 1:200 (ICC) | DSHB | Cat# H4B4 | AB_528129 |
| LC3B | 1:2500 (WB) | Sigma-Aldrich | Cat# L7543 | AB_796155 |
| p62 | 1:1000 (WB) | MBL | Cat# PM045 | AB_1279301 |
| β-Actin-HRP | 1:5000 (WB) | SCB | Cat# sc-47778 | AB_2714189 |
| Table S2b  List of antibodies and dilution used | | | | |
| **ANTIBODIES** | **DILUTION** | **SOURCE** | **IDENTIFIER** | **RRID** |
| NeuN | 1:150 (IHC) | Chemicon | Cat# MAB377 | AB_2298772 |
| Anti-mouse IgG, HRP-linked Antibody | 1:2500 | CST | Cat# 7076 | AB_330924 |
| Anti-rabbit IgG, HRP-linked Antibody | 1:2500 | CST | Cat# 7074 | AB_209923 |
| Anti-goat IgG, HRP-linked Antibody | 1:2500 | Biorad | Cat# 1721034 | AB_2617114 |
| Alexa Fluor® 647 Donkey anti-Goat IgG (H+L) | 1:500 | Invitrogen | Cat# A21447 | AB_2535864 |
| Alexa Fluor® 568 goat anti-mouse IgG (H+L) | 1:500 | Invitrogen | Cat# A11031 | AB_144696 |
| Alexa Fluor® 488 goat anti-mouse IgG (H+L) | 1:500 | Invitrogen | Cat#A11029 | AB_138404 |
| Alexa Fluor® 488 goat anti-rabbit IgG (H+L) | 1:500 | Invitrogen | Cat#A11034 | AB_2576217 |
| Alexa Fluor® 568 goat anti-rabbit IgG (H+L) | 1.500 | Invitrogen | Cat#A11036 | AB_10563566 |
| Alexa Fluor® 647 goat anti-rabbit IgG (H+L) | 1.500 | Invitrogen | Cat#A21244 | AB_2535812 |

IHC=Immunohistochemistry, ICC= Immunocytochmistry, WB= Western Blot, CST= Cell Signaling Technology, SCB=Santa Cruz Biotechnology, DSHB= Developmental Studies Hybridoma Bank, MBL= Medical and Biological Laboratories Co., Ltd.

Table S3a

Human tissue for AD and controls paraffin for IHC (hippocampus + medial temporal)

| **Age (years) / sex** | **Neuropathological diagnosis**  **(NIA-AA ABC score - (**[**Montine et al., 2012**](#_ENREF_4)**))** |
| --- | --- |
| 88 / F | Old small infarcts; no AD (A0 B0 C0) |
| 79 / M | Vascular disease; no AD (A1 B0 C0) |
| 76 / F | Acute brain trauma; no AD (A0 B0 C0) |
| 62 / F | Normal |
| 70 / F | Early Parkinson; no AD (A0 B0 C0) |
| 52 / M | Schizophrenia; early HI |
| 26 / M | normal |
| 85 / M | Alzheimer (A2 B3 C2) + vascular dementia |
| 75 / M | Early Alzheimer (A1 B1 C1) |
| 76 / M | Alzheimer (A3 B2 C3) with early dementia |
| 83 / M | Lewy body dementia + Alzheimer (A2 B2 C2 ) |
| 79 / F | Alzheimer (A3 B3 C3) with dementia |
| 70 / M | Severe Alzheimer (A3 B2 C3 ) + amyloid angiopathy |

Table S3b

Human brain samples used for Western blots

| **Age (years) / Sex** | **Neurological status** | **Post mortem time to freezing** |
| --- | --- | --- |
| 21 / F | Control | 34 hours |
| 45 / M | Control | 12 hours |
| 85 / M | AD | 4 hours |
| 83 / M | AD | 8 hours |
| 74 / M | AD | < 6 hours |
| 74 / F | AD | 5 hours |
| 55 / M | AD | 4 hours |
| 68 / F | Control | 11 hours |
| 75 / F | Control | 6 hours |
| 78 / M | AD | 4.5 hours |
| 86 / F | AD | 2 hours |
| 61 / M | AD | 5.5 hours |
| 74 / F | AD | 3.5 hours |
| 61 / M | Control | < 12 hours |
| 74 / F | Control | 3 hours |
| 65 / M | Control | Short |
| 75 / F | AD | 3.5 hours |

# **Figure Captions**

**Fig S1. CTSL mediated LB1 cleavage pattern is seen in APP/PS1 mouse brain.** (**A, B**) Expression of Aβ (determined with 6E10 antibody) significantly increases in 2 month and 3 month old 3xTg mouse hippocampus compared to their littermate control, but phosphorylation of tau was not significantly increased at this time point. Data are mean ± SEM, n=4, ** and *** refers to *P*<0.01 and *P*<0.001 respectively (t-test with their age matched control). (**B**) Western blot with hippocampal lysate from 3 month and 6 month APP/PS1 mouse showed 21 kDa fragment of LB1 and increased expression of CTSL compared to their age matched control mouse. (**C**) Densitometric quantification of cleaved LB1 (21 kDa) and CTSL (mean ± SEM, *n*=4-9, * and ** indicates *P*<0.05 and 0.01 respectively, t-test).

**Fig S2.** **LB1 invagination was not observed in astrocytes in human AD brain.** (**A**) Paraffin embedded hippocampal and medial temporal slides from CTL and AD patients were de-paraffinized and immuno-labeled for LB1 (green), GFAP (red) and DAPI (blue), scale bar = 10 µm). LB1 in astrocytes are less detected. (**B**) Fibrillogenesis of Aβ42 was determined using Thioflavin-T (Th-T) assay. 20 µM of Aβ42 was prepared in PBS and aliquot of 30 µl of the solution was incubated at 37 ° C for the indicated time period. 20 µl was taken from the reaction and mixed with 80 µl of 5 µM of Th-T in PBS. Fluorescence of the Aβ42 and Th-T mixture was measured using BioTek H1-Synergy microplate reader (Winnoski, VT, United States) at 440 nm of excitation and 490 nm of emission. Values are expressed as relative fluorescence unit (RFU). (C) SH-SY5Y cells were treated with Aβ42 (5 µM, 16 h) and cell lysates were used for western blotting for CASP9 and CASP8. No cleavage of CASP9 and CASP8 was observed in Aβ42 treated cells. β-Actin was employed as loading control.

**Fig S3. Differential cleavage of LB1 induced by Aβ42 and STS.** (**A**) Representative immunoblot examining the LB1 degradation product (SH-SY5Y cells) after exposure to STS (0.5 µM, 6h) and Aβ42 (5 µM, 16 h) using an anti-lamin B1 (N-terminal) antibody (CST#12586, RRID: AB_2650517). β-Actin was used as loading control. Despite robust decrease in pro-lamin B levels, no N-terminal fragment was detected in Aβ42 treated samples, while a 25 kDa N-terminal product was detected in STS treated samples. (**B**) SH-SY5Y cells were treated with 5 µM of Aβ42 for 16 h. Cells were then harvested, washed twice with ice cold PBS and underwent sub-cellular fractionation using digitonin buffer (sup) and NP-40 buffer (ppt) without sonication. Appearance of cleaved lamin B1 (21 kDa) in digitonin fragments (sup) only in Aβ42 treated cells not in control cells. In 1% NP-40 fraction without sonication (ppt), pro-lamin B1 was present in control cells, whereas, there was complete loss of pro-lamin B1 and appearance of cleaved lamin B1 (21 kDa) in Aβ42 treated cells. Histone H3 cleavage was observed only in Aβ42 treated precipitant not in control cell. Actin was used loading control. Data is representative of four independent experiments. (**C**) After sonication (5s x 3), fragment of control and Aβ42 treated cells had comparable pro-lamin B1 levels, representing the membrane-bound lamin-B1. Methylene blue was used to show the protein loading. (**D, E**) Purified rh-LB (1 µg) was incubated with increasing concentration of rhCTSL (3.12 – 200 ng) for 30 min at 37 °C. The reaction mixture was then analyzed by western blots using C-terminal LB1 antibody. CTSL inhibitor z-FY-CHO inhibited rhCTSL mediated rhLB1 cleavage whereas rCTSB did not cleave rhLB1. (**F**) Densitometry analysis of western blot in rat hippocampal neurons described in Fig. 4D.

**Fig S4.** **Lysosomal enlargement in Aβ42 toxicity.** (**A**) Immunolabeled 3D confocal micrographs depicting the size and distribution of CTSL in SH-SY5Y cells treated with Aβ42 (5 µM, 16 h) Scale bar = 10 µm. Size distribution of CTSL-positive and LAMP2-positive particles were performed using ImageJ and the results were categorized as small (0-0.499 µm^2^), medium (0.5-0.799 µm^2^) and large (0.8-1.5 µm^2^). (**B**) A significant decrease in small and increase in large particles was observed in Aβ42 treated group. Data are expressed as mean ± SEM, *n*=3 independent experiments. A minimum of 431 cells from each study/condition/group were used for quantification. * *P*<0.05. (**C**) Sub-cellular fractionation after treating SH-SY5Y cells with 5 µM of Aβ42 for 16 h showed that nuclear enriched fractions of Aβ42 treated cells contain increased amount of mature CTSL compared to control cells. (**D**) Western blot with conditioned media showed increased amount CTSL release in Aβ42 treated culture media compared to vehicle treated culture media. Ponceau R staining employed as loading control. Data are representative of 3 independent experiments.

**Fig S5.** **Cell-free enzymatic assay confirming the specificity of** **LB1 as specific substrate of CTSL.** (**A, B**) Whole cell extract of healthy SH-SY5Y cells was incubated with rhCTSL (50 ng) for 30 min at 37 °C. The reaction mixture was then analyzed by western blots using C-terminal LB1, CASP6 and histone H3 antibodies. The results recapitulated the Aβ42 treated cells. Data presented is representative of 4 independent experiments. (**C**) Nuclei enriched fraction from healthy SH-SY5Y cells were incubated in reaction buffer (pH 7.4) at 37 °C for 1 h with/without the indicated inhibitors. The reaction was stopped by adding 2x Laemmli buffer and was resolved by 12% SDS-PAGE and probed for LB1 cleavage by western blot. Representative blot of 4 independent experiment is shown. Only CTSL inhibitors effectively alleviated the cleavge of 21kDa LB1. (**D, E**) Western blot showing decreased level of CASP6 and Cystatin B in human hippocampal tissue. Data indicates mean ± SEM, *n*=3, ** and * indicates *P*<0.01 and 0.05, respectively, t-test. (**F**) MEF cells obtained from the wildtype (WT), CTSL^-/-^ and CTSB^-/-^ mice were subjected to western blot to confirm the complete deletion of CTSL and CTSB. (**G**) CTSL and CTSB enzymatic activity determined by using their specific synthetic substrates in cathepsin knocked out MEF cells. WT, Ctsl^-/-^ and Ctsb^-/-^ indicates wild type, CTSL and CTSB knock out, respectively. N.D = not detected. (**H**) MEF cells overexpressing human CTSL (TghCTSL^+/+^) and CTSB (TghCTSB^+/+^) were cultured and protein expression was confirmed by immunoblot. (**I**) CTSL and CTSB enzymatic activity for WT, Ctsl^+/+^ (human CTSL overexpressing) and Ctsb^+/+^ (hCTSB overexpressing) MEF cells.

**Fig S6.** **Alteration of DNA acetylation and methylation in hippocampal tissue in 3xTg mouse and human hippocampus.** (**A**) Representative western blots of key epigenetic modifiers in 3xTg mouse hippocampi and their age-matched controls and their (**B**) quantification. A minimum of 3 mice was used/experimental group and the data are presented as mean ± SEM. **P*<0.05 and **P<0.01. (**C, D**) Western blot and densitometric analysis of human hippocampal samples for indicated proteins. * indicates *P<* 0.05. (**E**) Mouse cortical neurons were treated with Aβ42 in presence or absence of CTSL inhibitors and immunolabelled with lamin B (green), H3K9ac (red) and DAPI. Signal intensity of H3K9ac for each cell was determined with ImageJ software and presented as scatter plot. Data = mean ± SEM, *n*=2, **** and ** indicates *P*<0.0001 and *P*<0.01 respectively.
